# Oxidative DNA damage drives apoptotic photoreceptor loss in *NMNAT1*-associated inherited retinal degeneration: a therapeutic opportunity

**DOI:** 10.1038/s41419-026-08680-7

**Published:** 2026-04-02

**Authors:** Hanmeng Zhang, Kevin Valestil, Rossano M. Butcher, Eric A. Pierce

**Affiliations:** Ocular Genomics Institute, Department of Ophthalmology, Massachusetts Eye and Ear, Harvard Medical School, Boston, MA USA

**Keywords:** Cell death, DNA adducts, Mechanisms of disease

## Abstract

Early-onset inherited retinal degenerations (IRDs), such as Leber congenital amaurosis (LCA) caused by pathogenic variants in the *NMNAT1* gene, lead to severe vision loss in children. Despite its ubiquitous expression, reduced *NMNAT1* function primarily affects photoreceptor cells (PRs) of the retina, yet the mechanisms underlying their vulnerability remain incompletely understood. Here, we demonstrate that reduced NMNAT1 enzyme function due to the p.V9M mutation leads to DNA damage in PRs, characterized by the progressive accumulation of the oxidative DNA adduct 8-oxo-dG in *Nmnat1*^V9M/V9M^ mutant mice. Cells with oxidative DNA damage also demonstrate DNA double-strand breaks, as evidenced by co-staining with antibodies to phosphorylated H2AX (γH2A.X). This DNA damage correlates with apoptosis-driven PR degeneration, as evidenced by caspase-9 activation and TUNEL staining in the PRs of the *Nmnat1*^V9M/V9M^ mutant mice, while alternative cell death pathways such as necroptosis and parthanatos were not significantly activated. Treatment with the antioxidant N-acetylcysteine (NAC) reduced oxidative DNA damage and retinal immune responses, mitigated apoptosis, and preserved cone PRs. Longitudinal assessment via optical coherence tomography (OCT) and electroretinography (ERG) revealed sustained structural and functional protection of the retina in NAC-treated mice. These findings establish oxidative DNA damage as a key driver of PR degeneration in the *Nmnat1*^V9M/V9M^ model and highlight NAC’s potential as a therapeutic strategy for *NMNAT1*-associated IRD and potentially other IRDs in which oxidative DNA damage contributes to disease pathogenesis.

## Introduction

Severe early-onset inherited retinal degenerations (IRDs) are a diverse group of genetic disorders that cause progressive vision loss in children, significantly impacting their quality of life and underscoring the urgent need for effective therapies to preserve retinal function [1, 2]. This study focuses on a rare early-onset IRD caused by recessive mutations in the *NMNAT1* gene, which encodes the nuclear protein nicotinamide mononucleotide adenylyltransferase 1, essential for the nicotinamide adenine dinucleotide (NAD^+^) biosynthesis in the nucleus [3–6]. Although *NMNAT1* is ubiquitously expressed, mutations in this gene predominantly result in retina-specific phenotypes [3–6]. Among the various *NMNAT1* variants, a common one is p.Val9Met (p.V9M) [3, 7]. This mutation is associated with the typical clinical features of Leber congenital amaurosis (LCA), including macular atrophy, nystagmus, progressive loss of retinal function, and severe vision loss [3]. To model this condition, we developed *Nmnat1*^V9M/V9M^ mice, which recapitulate key aspects of the human disease [8]. These mice exhibit early-onset retinal degeneration, marked by rapid loss of photoreceptor cells (PRs) within two months, followed by gradual degeneration of the inner retina and retinal pigment epithelium (RPE) [8].

Despite the ubiquitous expression of *NMNAT1*, the p.V9M mutation causes a reduction in NAD^+^ levels specifically within the retina, explaining the retina-specific phenotype [9]. However, the reason(s) why PRs are the most vulnerable to loss of NMNAT1 function compared to other cells remain unclear. In the retinas of *Nmnat1*^V9M/V9M^ mutant mice we detected the activation of poly (ADP-ribose) polymerases (PARPs), DNA damage sensors critical for initiating NAD^+^-dependent DNA repair processes, specifically in the outer nuclear layer (ONL), where PRs are located [8]. Further studies confirmed the presence of DNA damage using comet assays and localized this damage to the PR layer using γH2A.X, a marker of double-strand DNA breaks [10]. On average, human cells experience approximately 70 000 DNA lesions per day, arising from processes such as DNA oxidation, alkylation, hydrolysis, and replication errors [11–15]. Among these, oxidative DNA damage is a major contributor, particularly in PRs, which have exceptionally high metabolic demands due to their role in phototransduction, thereby increasing their susceptibility to DNA oxidation [16–20].

Severe and irreparable DNA damage can trigger multiple cell death pathways in PRs, such as caspase-dependent apoptosis, PARP-mediated parthanatos, and RIPK1-mediated necroptosis [21–25]. When DNA damage exceeds repair capacity, it initiates apoptotic signaling through caspase-9, a key regulator of the intrinsic apoptosis pathway [26]. In parallel, excessive PARP activation in response to severe DNA damage can deplete NAD^+^ levels, triggering the release of apoptosis-inducing factor (AIF) from mitochondria [27, 28]. AIF translocates to the nucleus, causing large-scale DNA fragmentation and chromatin condensation, resulting in parthanatos, a caspase-independent form of cell death [27, 28]. Additionally, DNA damage can trigger a signaling cascade where RIPK1 interacts with RIPK3 and MLKL to form the necrosome complex, leading to necroptosis, a regulated form of necrotic cell death characterized by plasma membrane rupture, release of intracellular contents, and inflammation [29–31].

In the *Nmnat1*^V9M/V9M^ mice, identifying the primary driver of DNA damage and the predominant cell death mechanisms in PRs is crucial for understanding the underlying mechanisms of PR degeneration and developing therapeutic approaches. In this study, we demonstrate that reduced NMNAT1 function in the *Nmnat1*^V9M/V9M^ mice results in the accumulation of oxidative DNA damage in PRs, marked by the accumulation of 8-oxo-dG and colocalization with γH2A.X. This damage correlates with apoptosis-driven PR degeneration. Treatment with the antioxidant N-acetylcysteine (NAC) significantly reduced oxidative DNA damage, mitigated retinal immune responses and apoptosis, and provided sustained structural and functional protection. These findings provide valuable insights into the protective mechanisms of antioxidants in PRs and identify oxidative DNA damage as a promising therapeutic target for *NMNAT1*-associated IRD and potentially other genetic forms of retinal degeneration.

## Results

### Oxidative DNA damage accumulates in the PRs of *Nmnat1*^V9M/V9M^ mutant mice

**Fig. 1:**
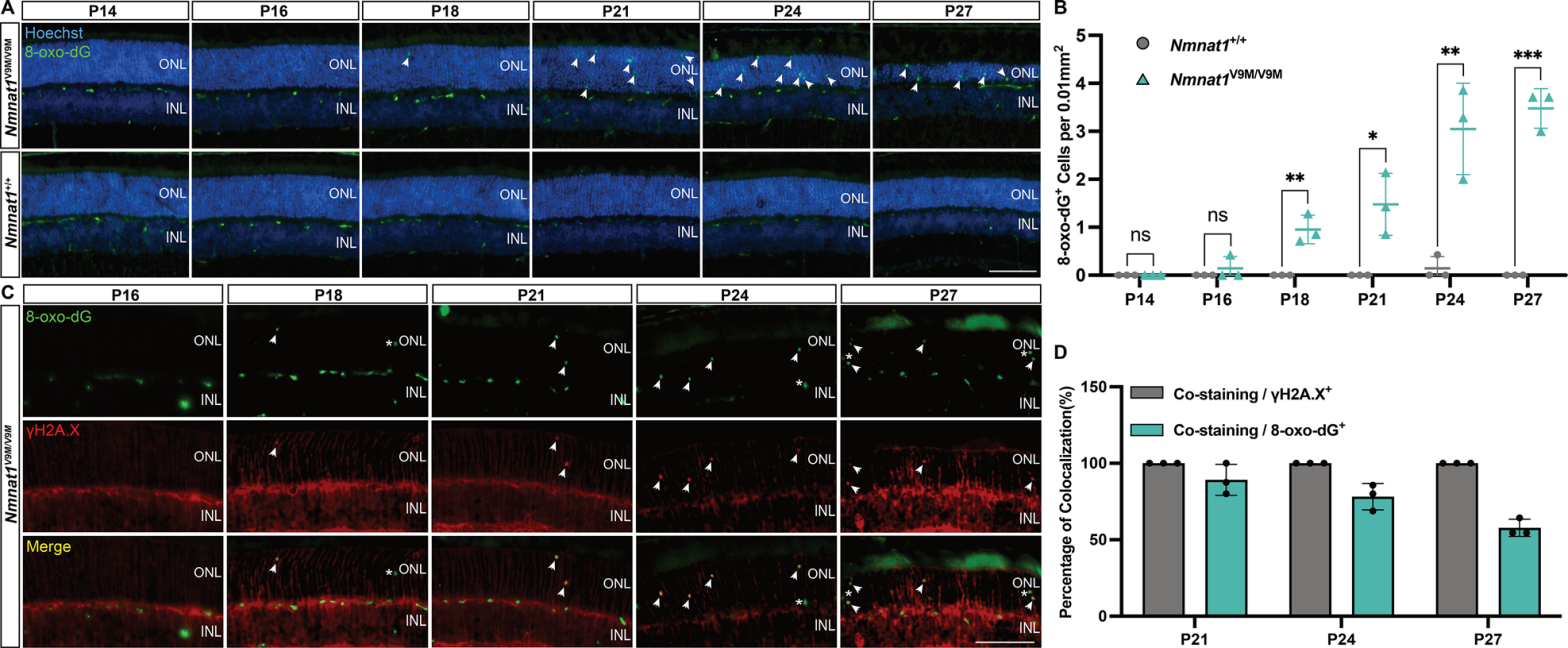
Accumulation of oxidative DNA damage in the *Nmnat1*^V9M/V9M^ mouse retina. **A** Representative immunofluorescence images showing the detection of 8-oxo-dG (green), a marker of oxidative DNA damage, in the ONL of the retinas of *Nmnat1*^V9M/V9M^ mutant mice. Nuclei are counterstained with Hoechst (blue). **B** Quantification of 8-oxo-dG levels in the ONL reveals a time-dependent increase in oxidative DNA damage in *Nmnat1*^V9M/V9M^ retinas (green triangle) compared to *Nmnat1*^+/+^ controls (gray circle). Significant accumulation is observed at P18, P21, P24, and P27. **C** Colocalization analysis of 8-oxo-dG (green) with the DNA double-strand break marker γH2A.X (red) in the ONL of *Nmnat1*^V9M/V9M^ retinas. Arrows indicate cells positive for both markers, while asterisks denote 8-oxo-dG-positive cells lacking γH2A.X signal. **D** Quantification of colocalization shows that all γH2A.X-positive cells (gray) also express 8-oxo-dG, while a subset of 8-oxo-dG-positive cells (green) do not colocalize with γH2A.X, suggesting oxidative DNA damage without detectable DSB formation in certain cells. Data are presented as mean ± standard deviation. Statistical comparisons between *Nmnat1*^V9M/V9M^ and *Nmnat1*^+/+^ groups were performed using multiple *t*-tests. *n* = 3 biological replicates per group. ***p < 0.001, **p < 0.01, *p < 0.05; ns, non-significant. Scale bar = 100 µm.

To determine whether DNA oxidation is the primary contributor to the DNA damage observed in the *Nmnat1*^V9M/V9M^ mutant mouse retina, we measured the oxidative DNA lesion 8-oxo-dG, a hallmark of DNA oxidation from P14 to P27, which spans the initial stage prior to and during PR degeneration [32]. Our analysis revealed a progressive accumulation of 8-oxo-dG in *Nmnat1*^V9M/V9M^ retinas compared to *Nmnat1*^+/+^ controls, with the first detection at P16 and a significant increase at later time points (P18, *p* = 0.0052; P21, *p* = 0.0165; P24, *p* = 0.0069; P27, *p* = 0001, (Fig. 1A, B). Double staining for 8-oxo-dG and γH2A.X showed that all γH2A.X-positive cells also expressed 8-oxo-dG (Fig. 1C, D), indicating that oxidative DNA damage is the primary endogenous source of DNA breaks in this model [32, 33]. Interestingly, a subset of 8-oxo-dG-positive cells (P21, 10.83%; P24, 21.85%; P27, 42.21%) lacked γH2A.X staining (Fig. 1C, asterisk; Fig. 1D), suggesting that oxidative DNA damage in these cells may not yet have led to detectable DNA strand breaks.

### Oxidative DNA damage correlates with PR apoptosis in the retinas of *Nmnat1*^V9M/V9M^ mutant mice

**Fig. 2:**
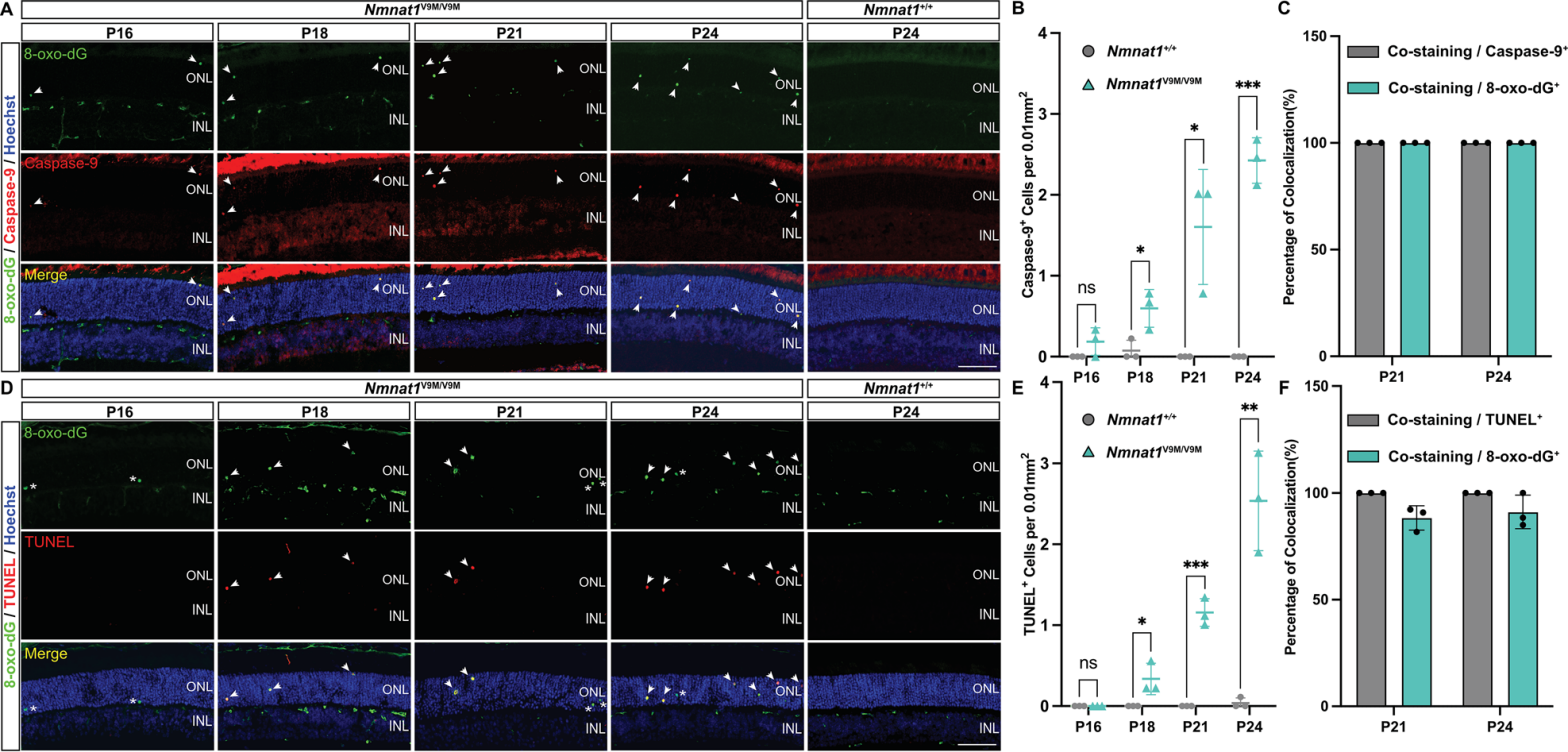
Association of oxidative DNA damage with apoptosis in the *Nmnat1*^V9M/V9M^ mouse retina. **A** Representative immunofluorescence images showing colocalization of 8-oxo-dG (green) with caspase-9 (red) in the retinas of *Nmnat1*^V9M/V9M^ mutant mice from P16 to P24. Arrows indicate cells positive for both markers. Nuclei are counterstained with Hoechst (blue). **B** Quantification of caspase-9-positive cells reveals a time-dependent increase in apoptosis in the *Nmnat1*^V9M/V9M^ retinas (green triangle) compared to *Nmnat1*^+/+^ controls (gray circle), with significant elevations at P18, P21, and P24. **C** Colocalization analysis showed that all caspase-9 signals overlap with 8-oxo-dG. **D** Representative images showing colocalization of 8-oxo-dG (green) with TUNEL staining (red) in the retinas of *Nmnat1*^V9M/V9M^ mutant mice from P16 to P24. Arrows highlight cells positive for both markers, while asterisks denote 8-oxo-dG-positive cells lacking TUNEL expression. **E** Quantification of TUNEL-positive cells demonstrates a progressive increase in apoptosis in the *Nmnat1*^V9M/V9M^ retinas (green triangle) compared to controls (gray circle), with significant differences at P18, P21, and P24. **F** Colocalization analysis reveals that all TUNEL signals overlap with 8-oxo-dG (gray), while a subset of 8-oxo-dG-positive cells (green) did have TUNEL staining. Data are presented as mean ± standard deviation. Statistical comparisons between *Nmnat1*^V9M/V9M^ and *Nmnat1*^+/+^ groups were performed using multiple *t*-tests. *n* = 3 biological replicates per group. ***p < 0.001, **p < 0.01, *p < 0.05; ns non-significant. Scale bar = 100 µm.

We assessed PR loss by quantifying the thickness of the Hoechst-positive PR nuclear layer across disease progression and observed a significant 25.13% decrease beginning at P24 (*p* = 0.0088), which progressed to a 39.57% decrease by P27 (*p* = 0.0029; Supplementary Fig. 1). To determine whether oxidative DNA damage correlates with PR loss, we first examined the cell death pathways activated in the retinas of *Nmnat1*^V9M/V9M^ mutant mice. Caspase-9, the initiator caspase of the intrinsic apoptotic pathway, was detected in PRs beginning at P16, followed by positive TUNEL staining at P18, indicating late-stage apoptosis (Fig. 2A, B, D, E). Both caspase-9 and TUNEL signals progressively increased over time, coinciding with the progression of PR degeneration (Fig. 2A, B, D, E) [26].

To assess the potential involvement of alternative cell death pathways, we analyzed markers of necroptosis and parthanatos [23–25]. Phospho-MLKL (S345), a marker of necroptosis, was not detected in the retinas of *Nmnat1*^V9M/V9M^ mutant mice (Supplementary Fig. 2A) [24, 25]. Additionally, while AIF expression was observed at P24 and increased further by P27, no mitochondrial-to-nuclear translocation was detected (Supplementary Fig. 2B, C), suggesting a cellular stress response rather than active parthanatos [27, 28].

To further investigate the relationship between oxidative DNA damage and apoptotic cell death, we performed double staining for 8-oxo-dG alongside apoptotic markers from P16 to P24 (Fig. 2A, B, D, E). While a subset of 8-oxo-dG-positive cells lacked TUNEL staining (Fig. 2D, asterisk; Fig. 2F), suggesting the possibility of DNA repair before progression to apoptosis, caspase-9 and TUNEL signals consistently colocalized with 8-oxo-dG at all time points (Fig. 2A, C, D, F). These findings establish a strong correlation between oxidative DNA damage and apoptosis, reinforcing the role of oxidative DNA damage in PR degeneration in the *Nmnat1*^V9M/V9M^ mouse model.

### NAC treatment reduces oxidative DNA damage and preserves PRs in the retinas of *Nmnat1*^V9M/V9M^ mutant mice

**Fig. 3:**
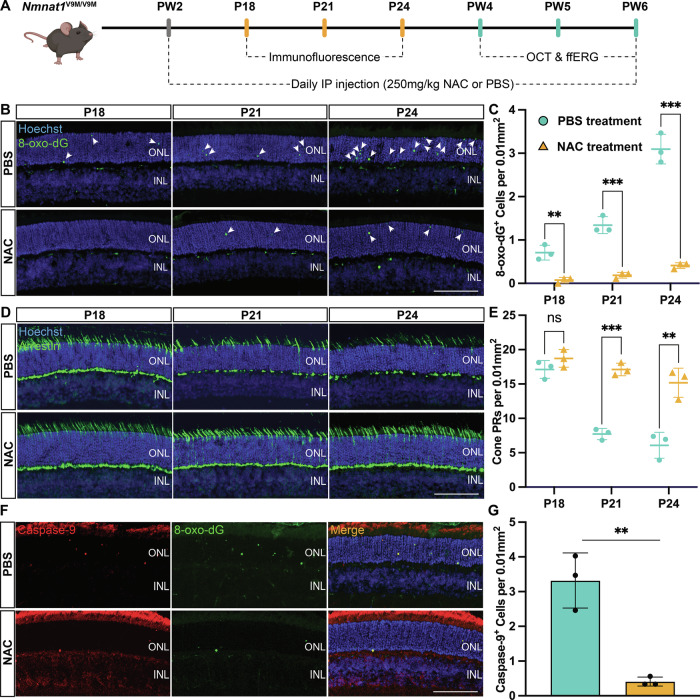
NAC treatment reduces oxidative DNA damage, mitigates apoptosis, and preserves cone photoreceptors in the *Nmnat1*^V9M/V9M^ mouse retina. **A** Flowchart of the experimental design for NAC treatment. NAC administration began at PW2 via daily intraperitoneal injections. Immunofluorescence analyses were conducted from postnatal days P18 to P24, while retinal structure and functional assays were performed from PW4 to PW6. **B** Representative immunofluorescence images showing reduced 8-oxo-dG signals (green, arrows) in the NAC-treated group compared to the PBS control. Nuclei are counterstained with Hoechst (blue). **C** Quantification of 8-oxo-dG levels demonstrates that NAC treatment significantly reduces oxidative DNA damage in the *Nmnat1*^V9M/V9M^ mouse retina at P18, P21, and P24 (orange triangle) compared to the PBS-treated control (green circle). **D** Representative images showing increased cone arrestin signals (red) in the NAC-treated group, indicating preserved cone PRs. **E** Quantification of cone arrestin-positive cells reveals a significant increase in the NAC-treated *Nmnat1*^V9M/V9M^ mouse retina at P21 and P24 (orange triangle) compared to the PBS-treated control (green circle). **F** Representative immunofluorescence images showing reduced caspase-9 signals (red) in the NAC-treated group at P21, with colocalization to 8-oxo-dG (green), suggesting mitigation of apoptosis. **G** Quantification confirms a significant reduction in caspase-9-positive cells following NAC treatment. Data are presented as mean ± standard deviation. Statistical comparisons between NAC-treated and PBS-treated *Nmnat1*^V9M/V9M^ groups were performed using multiple *t*-tests. *n* = 3 biological replicates per group. ***p < 0.001, **p < 0.01, *p < 0.05; ns non-significant. Scale bar = 100 µm.

We next investigated whether antioxidant treatment could mitigate oxidative DNA damage using N-acetylcysteine (NAC), a well-established antioxidant in retinal degeneration research [34, 35]. NAC treatment was initiated at postnatal week 2 (PW2), slightly earlier than the observed increase in 8-oxo-dG levels (P18), ensuring antioxidant activity prior to the onset of oxidative DNA damage accumulation (Fig. 1). The effects of daily NAC administration were evaluated at P18, P21, and P24, using PBS-treated *Nmnat1*^V9M/V9M^ mice as controls (Fig. 3A). NAC treatment significantly reduced the number of 8-oxo-dG-positive cells in the retinas of *Nmnat1*^V9M/V9M^ mutant mice at all examined time points (P18: 89.47%, *p* = 0.0039; P21: 86.08%, *p* = 0.0006; P24: 86.76%, *p* = 0.0002; Fig. 3B, C).

Given that *Nmnat1*^V9M/V9M^ mice exhibit early cone PR loss, mirroring the clinical presentation of IRD caused by *NMNAT1* mutations, we next sought to determine whether reducing oxidative DNA damage with NAC treatment could decrease cone PR death [10]. NAC-treated *Nmnat1*^V9M/V9M^ mice exhibited significant cone PR preservation, as indicated by the cone marker arrestin, at all examined time points: P18 (1.09-fold, *p* = 0.2022), P21 (2.22-fold, *p* = 0.0002), and P24 (2.5-fold, *p* = 0.0051) (Fig. 3D, E). We also investigated whether the reduction of oxidative DNA damage was accompanied by decreased apoptosis and found an 88% reduction of colocalized caspase-9 and 8-oxo-dG staining following NAC treatment (*p* = 0.0033; Fig. 3F, G).

### NAC treatment provides sustained protective effects on retinal structure and function

**Fig. 4:**
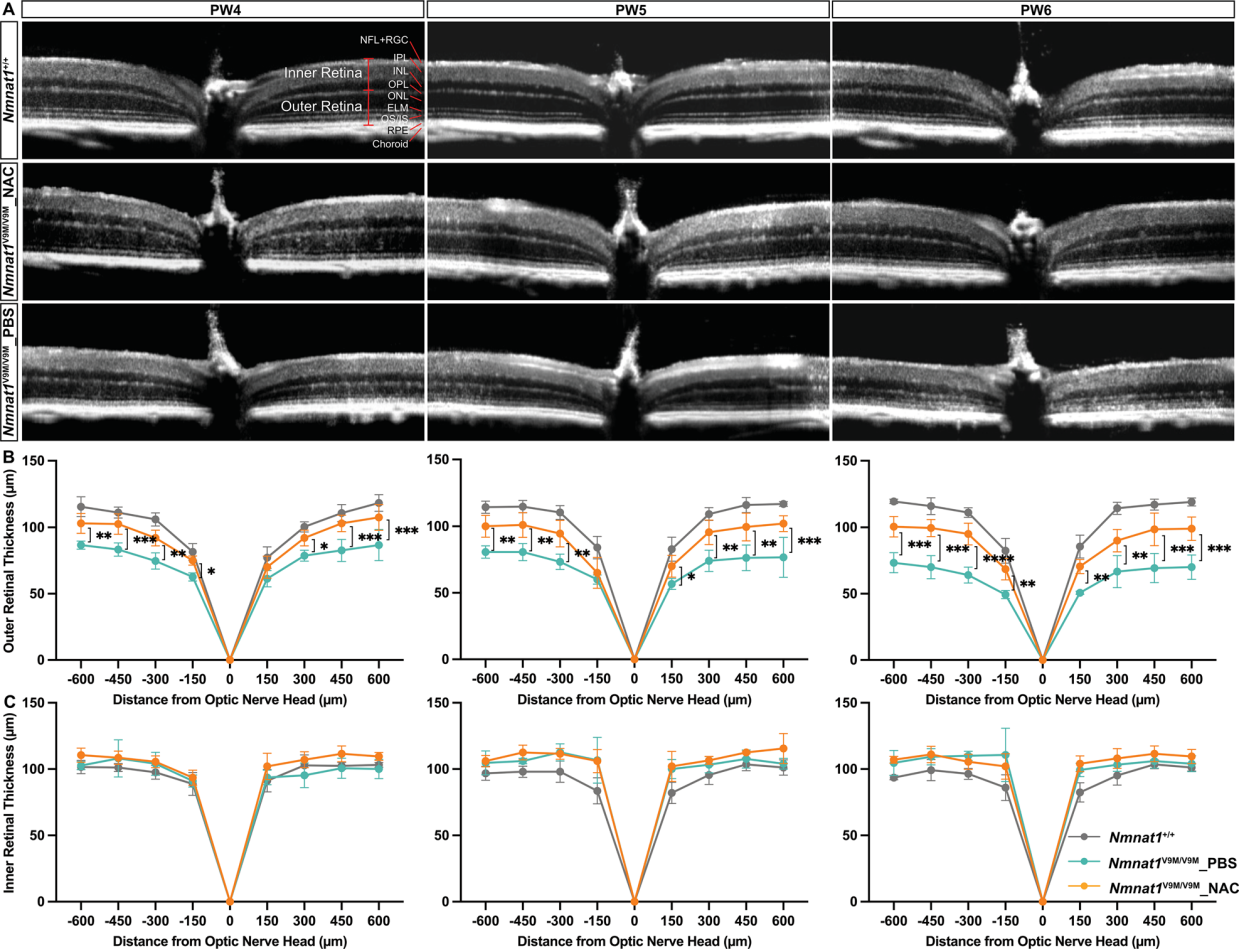
Retinal structure of NAC- and PBS-treated *Nmnat1*^V9M/V9M^ mouse retina. **A** Representative OCT scans from one eye of untreated *Nmnat1*^+/+^ (top panel), NAC-treated *Nmnat1*^V9M/V9M^ (middle panel), and PBS-treated *Nmnat1*^V9M/V9M^ mice (bottom panel) at PW4, PW5, and PW6. **B** Quantification of outer retinal thickness shows that NAC-treated *Nmnat1*^V9M/V9M^ mice (green) exhibit significant thinning compared to PBS-treated *Nmnat1*^V9M/V9M^ mice (orange) at PW4 (left panel), PW5 (middle panel), and PW6 (right panel). However, the outer retinal thickness in NAC-treated mice does not fully recover to the level of untreated *Nmnat1*^+/+^ mice (gray). **C** No significant changes were observed in inner retinal thickness among untreated *Nmnat1*^+/+^ (gray), NAC-treated *Nmnat1*^V9M/V9M^ (green), and PBS-treated *Nmnat1*^V9M/V9M^ mice (orange) at PW4 (left panel), PW5 (middle panel), and PW6 (right panel). NFL nerve fiber layer, RGC retinal ganglion cell, IPL inner plexiform layer, INL inner nuclear layer, OPL outer plexiform layer, ONL outer nuclear layer, ELM external limiting membrane, OS outer segment, IS inner segment, RPE retinal pigment epithelium. The inner retina includes layers from the inner edge of the NFL to the outer edge of the INL, while the outer retina includes layers from the inner edge of the OPL to the outer edge of the RPE. Data are presented as mean ± standard deviation. Statistical comparisons between groups were performed using two-way ANOVA followed by Tukey’s post hoc multiple comparisons test. *n* = 5 for *Nmnat1*^+/+^ mice group, *n* = 3 for PBS-treated *Nmnat1*^V9M/V9M^ mice group, *n* = 4 for NAC-treated *Nmnat1*^V9M/V9M^ mice group. ***p < 0.001, **p < 0.01, *p < 0.05. Multiple comparisons between *Nmnat1*^+/+^ and other groups, as well as non-significant changes, are not labeled.

In addition to assessing the early therapeutic effects of NAC to investigate disease mechanisms, we next examined whether prolonged NAC administration to PW6 could confer sustained structural and functional protection. OCT imaging revealed a significant and consistent increase in outer retinal thickness in NAC-treated *Nmnat1*^V9M/V9M^ mice compared to PBS-treated controls at all time points: 1.14- to 1.25-fold at PW4 (*p* = 0.0884–0.0003), 1.08- to 1.33-fold at PW5 (*p* = 0.4372–0.0008), and 1.35- to 1.48-fold at PW6 (*p* = 0.0049–<0.0001), consistent to histology findings (Supplementary Fig. 3), although this thickness did not fully return to that of *Nmnat1*^+/+^ controls (Fig. 4A, B, Supplementary Fig. 3). In contrast to the outer retina, inner retinal thickness showed no significant differences among *Nmnat1*^+/+^, PBS-treated *Nmnat1*^V9M/V9M^, and NAC-treated *Nmnat1*^V9M/V9M^ groups (Fig. 4A, C). This finding confirms that reduced NMNAT1 function primarily affects PRs in the outer retinal, with minimal impact on inner retinal neurons in the early stages of the disease [9, 10, 36, 37].

**Fig. 5:**
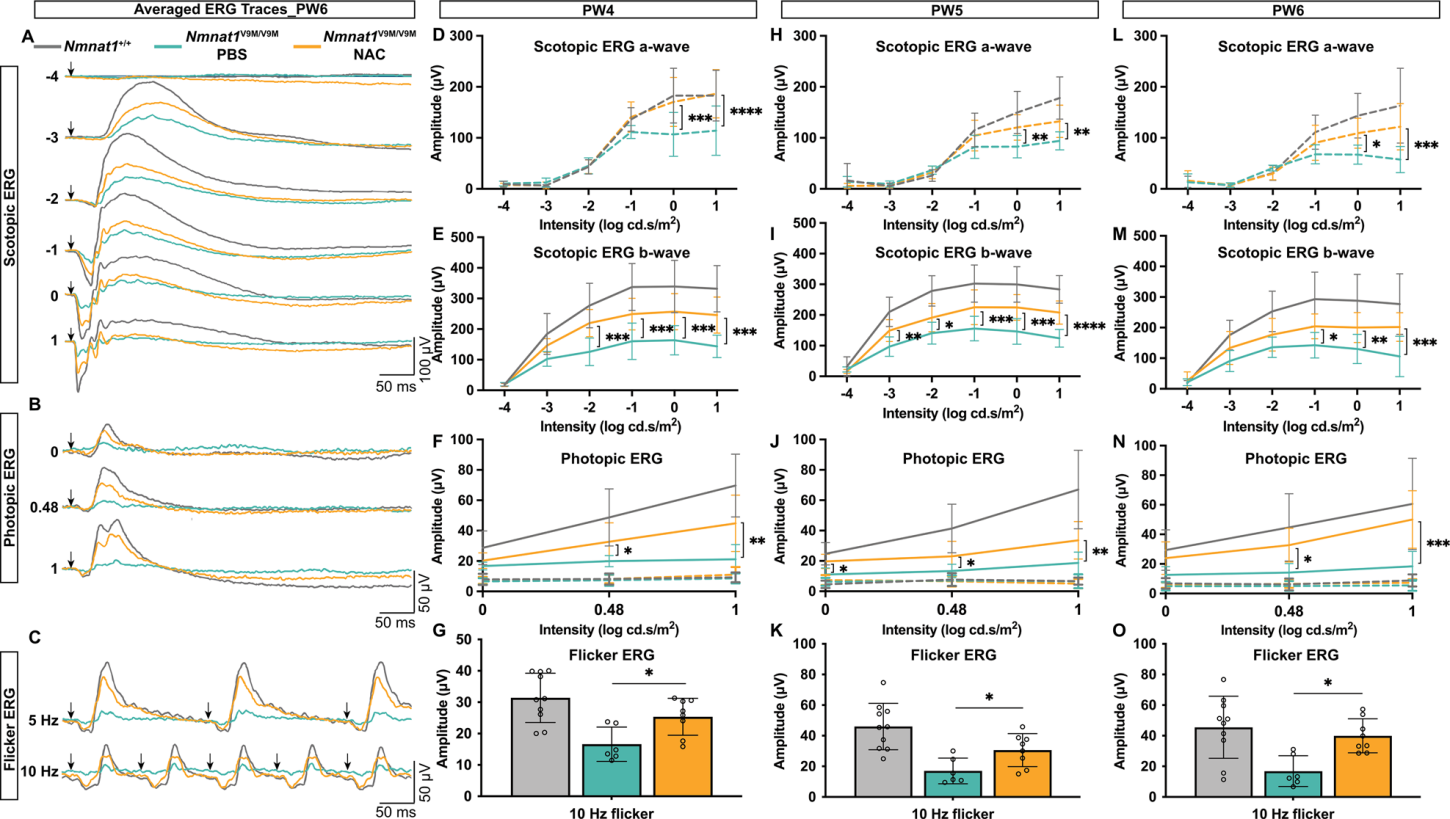
ERG analysis of NAC- and PBS-treated *Nmnat1*^V9M/V9M^ mouse retina. **A**–**C** Averaged ERG traces of untreated *Nmnat1*^+/+^ (gray), NAC-treated *Nmnat1*^V9M/V9M^ (orange), and PBS-treated *Nmnat1*^V9M/V9M^ mice (green) at PW6. Arrows indicate the timing of the stimulus flash. **D**, **E**, **H**, **I**, **L**, **M** Statistical analysis of dark-adapted, single-flash ERG responses showing b-wave (**D**, **H**, **L**) and a-wave (**E**, **I**, **M**) amplitudes in untreated *Nmnat1*^+/+^ (gray), NAC-treated *Nmnat1*^V9M/V9M^ (orange), and PBS-treated *Nmnat1*^V9M/V9M^ mice (green) at PW4 (**D**, **E**), PW5 (**H**, **I**), and PW6 (**L**, **M**). **F**, **J**, **N** Statistical analysis of b-wave amplitudes from light-adapted, single-flash ERG responses in untreated *Nmnat1*^+/+^ (gray), NAC-treated *Nmnat1*^V9M/V9M^ (orange), and PBS-treated *Nmnat1*^V9M/V9M^ mice (green) at PW4 (**F**), PW5 (**J**), and PW6 (**N**). **G**, **K**, **O** Statistical analysis of b-wave amplitudes from light-adapted flicker ERG responses at 5 Hz and 10 Hz in untreated *Nmnat1*^+/+^ (gray), NAC-treated *Nmnat1*^V9M/V9M^ (orange), and PBS-treated *Nmnat1*^V9M/V9M^ (green) at PW4 (**G**), PW5 (**K**), and PW6 (**O**). Data are presented as mean ± standard deviation. Statistical comparisons between groups were performed using two-way ANOVA for single-stimulus ERG analyses and one-way ANOVA for 10-Hz flicker ERG analyses, followed by Tukey’s post hoc multiple comparisons test. *n* = 5 for *Nmnat1*^+/+^ mice group, *n* = 3 for PBS-treated *Nmnat1*^V9M/V9M^ mice group, *n* = 4 for NAC-treated *Nmnat1*^V9M/V9M^ mice group. ***p < 0.001, **p < 0.01, *p < 0.05. Multiple comparisons between *Nmnat1*^+/+^ and other groups, as well as non-significant changes, are not labeled.

Consistent with the structural findings from OCT imaging, NAC treatment partially preserved retinal function in *Nmnat1*^V9M/V9M^ mice. The electroretinogram (ERG) responses of NAC-treated *Nmnat1*^V9M/V9M^ mice were improved compared to PBS-treated control mice at PW4, PW5, and PW6, although they remained below wildtype (WT, *Nmnat1*^+/+^) levels in most cases (Fig. 5). Under scotopic (rod-dominant) and photopic (cone-dominant) conditions, NAC-treated mice exhibited higher PR response amplitudes compared to PBS-treated *Nmnat1*^V9M/V9M^ controls (Fig. 5A, B, D–F, H–J, L–N). These functional improvements were maintained over the two-week evaluation period and were pronounced at PW6, a stage when PR degeneration is typically more severe. Specifically, NAC treatment led to a 0.73- to 2.12-fold increase in scotopic a-wave amplitudes (*p* = 0.9968–0.0002), a 1.23- to 1.91-fold increase in scotopic b-wave amplitudes (*p* = 0.7777–0.0002), and a 1.9- to 2.72-fold increase in photopic b-wave amplitudes (*p* = 0.1041–0.0002). In photopic flicker ERG, a test optimized to suppress rod activity and isolate cone function, response amplitudes were increased in NAC-treated *Nmnat1*^V9M/V9M^ mice compared to PBS-treated *Nmnat1*^V9M/V9M^ mice (PW4: 1.53-fold increase at 10 Hz, *p* = 0.0143; PW5: 1.8-fold increase at 10 Hz, *p* = 0.0467; PW6: 2.37-fold increase at 10 Hz, *p* = 0.0206 (Fig. 5C, G, K, O), further demonstrating improved cone function with a sustained effect.

### NAC attenuates cGAS-STING-independent retinal immune responses, which do not contribute to PR degeneration in the *Nmnat1*^V9M/V9M^ mutant mice

**Fig. 6:**
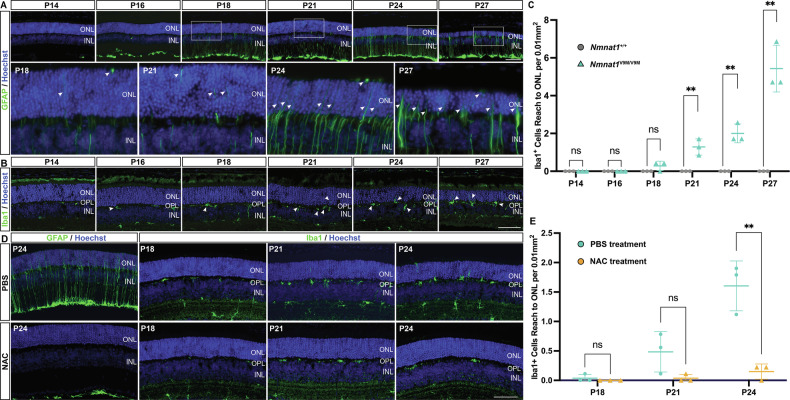
Progression of immune responses in the *Nmnat1*^V9M/V9M^ mouse retina and reduction by NAC treatment. **A** Representative images showing progressive upregulation of GFAP expression (red) in the *Nmnat1*^V9M/V9M^ retina from P14 to P27. White boxes in the top panel are magnified in the bottom panel. Arrows indicate GFAP-positive fibers extending into the ONL, with increasing intensity and distribution over time. Nuclei are counterstained with Hoechst (blue). **B** Representative images showing increased Iba1-positive microglia (green) and their migration to the ONL at later time points. Arrows highlight reactive microglia with hypertrophic morphology and their movement toward the ONL. **C** Quantification of Iba1-positive cells that have migrated to the ONL in *Nmnat1*^V9M/V9M^ (green triangle) and *Nmnat1*^+/+^ (gray circle) groups, demonstrating a significant increase in microglial migration in the *Nmnat1*^V9M/V9M^ retina. **D** Representative images showing reduced GFAP overexpression and decreased Iba1-positive microglial migration to the ONL in NAC-treated *Nmnat1*^V9M/V9M^ retinas compared to PBS-treated controls. **E** Quantification of Iba1-positive cells in the ONL confirms a significant reduction in microglial migration in NAC-treated *Nmnat1*^V9M/V9M^ retinas (orange triangle) compared to PBS-treated controls (green circle). Data are presented as mean ± standard deviation. Statistical comparisons between *Nmnat1*^V9M/V9M^ and *Nmnat1*^+/+^ groups or NAC-treated and PBS-treated *Nmnat1*^V9M/V9M^ groups were performed using multiple *t*-tests. *n* = 3 biological replicates per group. **p < 0.01, *p < 0.05; ns non-significant. Scale bar = 100 µm.

In addition to its well-documented antioxidant effects, NAC has also been shown to regulate immune responses [38, 39]. We therefore investigated whether its protective effect on PRs might also involve modulation of retinal immune responses [40–42]. To address this, we first characterized the time course of immune activation in the retinas of *Nmnat1*^V9M/V9M^ mutant mice from P14 to P27. Using glial fibrillary acidic protein (GFAP) as a marker for astrocytes and Müller cell processes, we observed a significant upregulation of GFAP in the *Nmnat1*^V9M/V9M^ retinas accompanied by the extension of GFAP-positive filaments from the inner to the outer retina starting at P18, a hallmark of gliosis (Fig. 6A) [43]. Iba1 staining also revealed reactive microglia, the resident immune cells of the retina, exhibiting hypertrophy starting at P18 (Fig. 6B) [44–46]. These reactive microglia accumulated in the OPL and migrated toward the ONL by P21 (P21, *p* = 0.0065; P24, *p* = 0.0022; P27, *p* = 0.0016, Fig. 6B, C). Following NAC treatment, both GFAP-marked gliosis and Iba1-marked microglial activation and migration were significantly reduced (Fig. 6D). At P24, there was a 90.7% decrease in Iba1-positive microglia reaching the ONL (*p* = 0.0047, Fig. 6E). These findings suggest that NAC effectively attenuates immune responses in the retinas of *Nmnat1*^V9M/V9M^ mice.

A key link between DNA damage and immune activation is the cGAS-STING signaling pathway [47]. In bulk RNA-sequencing analysis of the retinas of *Nmnat1*^V9M/V9M^ mutant mice, we observed significant upregulation of key components of this pathway, such as *Cgas* (2.2-fold), *Tmem173* (encoding STING, 2.8-fold), and Btk (4.3-fold) [10]. Immunofluorescence and western blot analyses further confirmed increased STING protein levels in the mutant retinas (Supplementary Fig. 4A, B). To assess whether cGAS-STING signaling links oxidative DNA damage to retinal immune activation, we generated *Nmnat1*^V9M/V9M^*:Sting*^-/-^ double mutant mice. Loss of STING did not reduce the number of CD11b-positive microglia/macrophages or the extent of GFAP-positive gliotic fibers extending into the PR layer (Supplementary Fig. 4C). Additionally, PR degeneration persisted in the double mutants, as assessed by OCT imaging (Supplementary Fig. 4D). These findings suggest that although the cGAS-STING pathway is activated in the *Nmnat1*^V9M/V9M^ mouse retina, its inhibition alone is insufficient to suppress immune activation or reduce PR degeneration in this model.

**Fig. 7:**
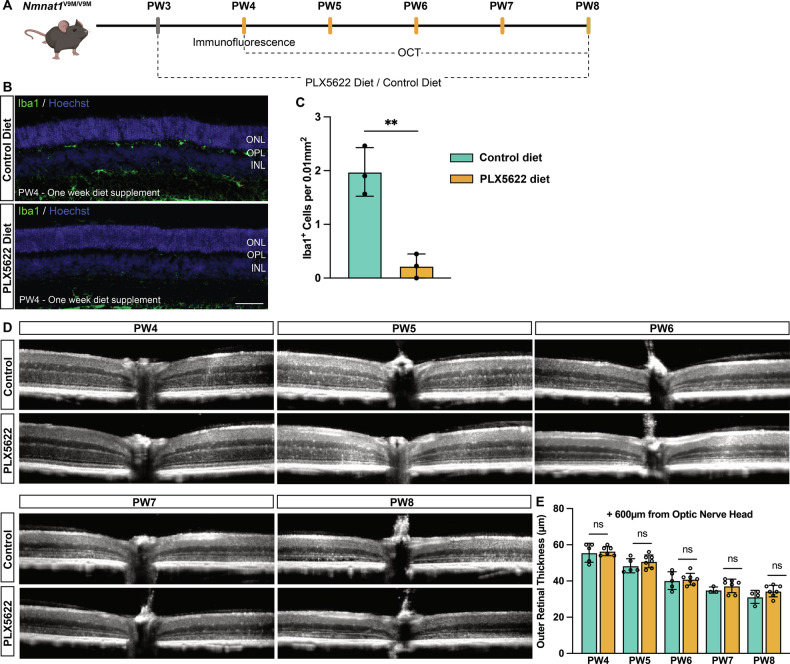
Microglia depletion does not significantly reduce PR degeneration in the *Nmnat1*^V9M/V9M^ mouse retina. **A** Flowchart of the experimental design for microglia depletion. Mice were fed a special diet containing PLX5622 or a control diet starting at PW3. Immunofluorescence was performed one week after diet initiation to assess microglia depletion efficiency. Retinal structure was evaluated in vivo from PW4 to PW8 to determine the protective effects of microglia depletion. **B** Representative immunofluorescence images of Iba1 (green) at one week post-diet supplementation. Markedly reduced reactive microglia were observed in the PLX5622-treated group, while control diet-treated mice showed accumulation of reactive microglia in the OPL and ONL at PW4. Nuclei are counterstained with Hoechst (blue). **C** Quantification of Iba1-positive cells reaching the ONL in PLX5622-treated (orange) and sham-treated (green) *Nmnat1*^V9M/V9M^ mouse retinas, confirming efficient microglia depletion in the PLX5622 group. **D** Representative OCT scans of PLX5622- and control diet-treated *Nmnat1*^V9M/V9M^ mouse retinas from PW4 to PW8. **E** Quantification of outer retinal thickness shows no significant difference between PLX5622-treated and sham-treated *Nmnat1*^V9M/V9M^ retinas, indicating that microglia depletion does not prevent PR degeneration. Data are presented as mean ± standard deviation. Statistical comparisons between PLX5622-treated and sham-treated *Nmnat1*^V9M/V9M^ groups were performed using multiple *t*-tests. *n* = 3 biological replicates per group for Iba1-positive cell quantification, and *n* = 6 mice per group for outer retinal thickness analysis. **p < 0.01; ns non-significant. Scale bar = 100 µm.

To further determine whether retinal immune responses contribute significantly to PR degeneration in this mouse model, we administered a diet containing PLX5622, a colony-stimulating factor 1 receptor (CSF1R) inhibitor, to deplete microglia [48–50]. The PLX5622 diet or a control diet was introduced at P21, coinciding with the onset of immune cell migration to the ONL (Figs. 6A–C and 7A). Microglial depletion efficiency was confirmed within one week of treatment, with a significant reduction in Iba1-positive cells (88.67%, *p* = 0.0038, Fig. 7B, C). However, OCT measurements of outer retinal thickness at PW4-8 showed no significant differences between PLX5622-treated and untreated groups (Fig. 7D, E). This suggests that retinal immune responses do not contribute significantly to PR degeneration in *Nmnat1*^V9M/V9M^ mouse retina.

## Discussion

Our studies investigated whether oxidative DNA damage serves as a primary driver of PR degeneration in the retinas of *Nmnat1*^V9M/V9M^ mutant mice and evaluated the therapeutic potential of mitigating such damage through antioxidant intervention. The results reported demonstrate progressive accumulation of oxidative DNA damage in PRs of *Nmnat1*^V9M/V9M^ mutant mice. Further, we found that 8-oxo-dG specifically colocalized with apoptotic markers rather than markers of other cell death pathways, indicating that oxidative DNA damage causes PR cell death via apoptosis in the *Nmnat1*^V9M/V9M^ mutant mice. Of particular note, treatment with the antioxidant NAC reduced oxidative DNA damage in PRs, leading to reduced cell death and sustained protection of PR structure and function. NAC treatment also attenuated retinal immune responses in the *Nmnat1*^V9M/V9M^ mice. However, this local immune response does not appear to play a major role in driving PR degeneration, as depleting microglia with PLX5622 did not alter the retinal degeneration phenotype [48–50]. Further, although the cGAS-STING pathway was found to be activated, it does not seem to link oxidative DNA damage to immune response in the *Nmnat1*^V9M/V9M^ mouse retina, since *Sting* knockout failed to reduce retinal immune activation. Overall, these results highlight oxidative DNA damage as a promising therapeutic target for *Nmnat1*-associated retinal degeneration.

8-oxo-dG is commonly used as a marker of oxidative DNA damage because guanine is the most susceptible nucleobase to oxidation [51, 52]. Under normal conditions, cells maintain minimal 8-oxo-dG levels through a tightly balanced system where oxidative stress generates it and DNA repair mechanisms clear it. In the retinas of *Nmnat1*^V9M/V9M^ mutant mice, the accumulation of 8-oxo-dG suggests a disruption of this balance. Consistent with our previous observation of no significant increase in the GSSG/GSH ratio, we did not detect lipid peroxidation in the mutant mouse retina (Supplementary Fig. 5A, B) [9]. These findings raise the possibility that impaired DNA repair, rather than oxidative stress, may contribute to the observed imbalance. NAD⁺ is a critical cofactor for enzymes involved in DNA repair, such as PARPs, sirtuins, and DNA ligases [9, 53]. Although direct evidence linking reduced NAD⁺ to impaired DNA repair in the retina is limited, studies in other tissues, such as skin and brain, support this connection [54, 55]. Future investigation of DNA repair in the *Nmnat1*^V9M/V9M^ mouse retina might provide mechanistic insight into oxidative DNA damage in PRs.

Interestingly, 8-oxo-dG accumulation is specifically localized to PRs in *Nmnat1*^V9M/V9M^ mutant mice, despite the ubiquitous expression of *Nmnat1*. Why are PRs particularly vulnerable? Unlike other retinal cells, PRs face amplified ROS generation due to intense oxygen consumption, light exposure, and oxidation-prone, polyunsaturated fatty acid-rich membranes, all of which heighten their reliance on robust DNA repair to counteract oxidative damage [16, 56, 57]. This unique vulnerability means that any disruption in the delicate balance between DNA oxidation and repair disproportionately affects PRs. Indeed, elevated 8-oxo-dG levels in PRs are not unique to the *Nmnat1* mutation but are also observed in other genetic forms of IRD and light-induced retinal damage, suggesting that oxidative DNA damage is a common pathological hallmark across diverse forms of PR degeneration [58, 59].

We observed a strong correlation between oxidative DNA damage and apoptosis. This aligns with established evidence that DNA damage triggers apoptotic PR death, as observed in the light-induced PR degeneration model, and supported by RNA-seq data showing enrichment of apoptotic pathways in the retinas of *Nmnat1* mutant mice [10, 21]. In particular, we detected the activation of caspase-9, a hallmark of the intrinsic apoptotic pathway typically initiated by cellular stressors such as DNA damage and oxidative stress [26]. Although our earlier work revealed PARP activation and reduced NAD⁺ levels, which are typically associated with parthanatos, a PARP-dependent cell death pathway, AIF activation without translocation from mitochondria to nucleus likely reflects a mitochondrial stress rather than contributing to parthanatos, indicating apoptosis appears to be the predominant mechanism driving PR degeneration in *Nmnat1*-associated IRD [27, 28].

As an antioxidant, NAC functions both by directly scavenging ROS and serving as a precursor for glutathione synthesis, making it highly effective in models of oxidative stress-driven PR degeneration, such as retinitis pigmentosa and light-induced damage [35, 60]. However, in *Nmnat1* mutant mice, NAC treatment did not alter lipid peroxidation markers (Supplementary Fig. 5A, B), nor did it directly increase retinal NAD⁺ levels (Supplementary Fig. 5C). These findings suggest that NAC may help rebalance the oxidative DNA damage-repair equilibrium by lowering basal ROS burden and thereby reducing the demand on DNA repair systems. This hypothesis implies NAC may have broader therapeutic potential, including in other forms of IRDs in which oxidative stress is not a primary driver, but oxidative DNA damage contributes to disease pathogenesis [59, 61].

In addition to its protective effects in the *Nmnat1*-V9M mouse retina, NAC treatment did not alter normal retinal structure (Supplementary Fig. 6A, B), body weight (Supplementary Fig. 6C), or cause liver and kidney toxicity in WT mice (Supplementary Fig. 6D), supporting its preclinical safety. The NAC dose used in our mouse study corresponds to ~20 mg/kg in humans (~1–1.5 g/day for a 70 kg adult) based on body surface area conversion [62]. The ongoing Phase 3 “NAC Attack” trial (NCT05537220) is evaluating long-term NAC treatment in adults with rod-cone degeneration (also called retinitis pigmentosa) at 3.6 g/day over 45 months, with safety closely monitored, and the results are expected to provide valuable insight into the translational potential of long-term NAC therapy [63]. However, NAC’s anti-apoptotic properties could theoretically allow the survival of cells that should otherwise be eliminated, especially critical during development, so its use in children requires additional caution [64]. Several clinical trials have tested the safety of NAC treatment in pediatric populations with doses ranging from 0.9 to 2.7 g/day, but not specifically for retinal degeneration, and were also limited to short-term treatment (4–12 weeks) [65–67]. Long-term studies of NAC in juvenile animal models and pediatric patients, with careful monitoring of retinal and systemic development, organ function, and potential toxicity, will be needed to assess safety.

It is not clear why cones degenerate earlier than rods in *Nmnat1*-associated IRD [4, 36]. One possibility is that cones are more sensitive to NAD^+^ reduction because they rely more on glycolysis, which uses NAD⁺ as an electron acceptor to support rapid cytosolic ATP production [68]. This allows cones to quickly adapt to changes in light levels, whereas rods depend more on oxidative phosphorylation, which generates larger amounts of ATP to sustain the dark current [68]. Consistent with the differences between rods and cones, we observed that the protective effect of NAC is more pronounced in cones, which exhibited a greater improvement in ERG responses, showing a 1.9- to 2.72-fold increase in photopic b-wave amplitudes, compared to a 1.23- to 1.91-fold increase in scotopic b-wave amplitudes. The possibility that rods and cones have distinct responses to NMNAT1 deficiency warrants further investigation, possibly using single-cell approaches [69].

While the retina is an immune-privileged tissue, immune cell activation can occur frequently as a common response to retinal damage arising from various causes [70, 71]. In our study, NAC also limited retinal immune responses (Fig. 6A–C). However, depletion of immune cells alone does not confer the same protective effect as NAC (Fig. 7D, E). These findings could be attributed to two potential explanations. First, retinal immune activation follows a biphasic pattern, with early anti-inflammatory or neuroprotective responses and a later shift toward a pro-inflammatory, neurotoxic phenotype under sustained stress or damage [72, 73]. Consistent with this, microglial depletion has been shown to either rescue PRs or worsen PR loss depending on the disease model and timing [50, 74]. In our study, microglia depletion initiated at PW3 did not produce significant benefit or harm to PRs in the *Nmnat1*^V9M/V9M^ model, suggesting a balanced anti-inflammatory and pro-inflammatory role of microglia during the early stage of immune response. It is possible that this intervention did not capture pathogenic phases of microglial involvement. Further studies will be required to more precisely define the temporal role of microglia in *NMNAT1*-associated retinal degeneration and to determine the optimal timing for therapeutic modulation. Second, PR degeneration in the *Nmnat1*^V9M/V9M^ model is likely driven primarily by oxidative DNA damage, with immune activation as a secondary consequence of such damage that does not contribute much to PR loss. We observed that the onset of retinal immune responses coincided with the appearance of oxidative DNA damage at P18, whereas immune cell migration to the ONL occurred slightly later, around P21 (Figs. 1 and 6A–C). Although we observed activation of the cGAS-STING pathway in the *Nmnat1*^V9M/V9M^ retina, genetic ablation of *Sting* did not reduce retinal immune responses. This suggests that the cGAS-STING pathway may not play a dominant role in linking oxidative DNA damage to immune activation in this context. Other DNA-sensing pathways, such as the AIM2 and NLRP3 inflammasomes, may be involved and need further investigation [75, 76].

Together, our findings demonstrate that oxidative DNA damage is a key driver of apoptosis in *Nmnat1*^V9M/V9M^ PR degeneration. Treatment with NAC effectively reduces oxidative DNA damage and preserves PRs. Future studies could employ single-cell RNA sequencing approaches to investigate whether DNA repair pathways are disrupted in *Nmnat1*^V9M/V9M^ mouse PRs, what drives early degeneration in cones, and how oxidative DNA damage is linked to retinal immune responses. Understanding these mechanisms may help guide new treatments for oxidative DNA damage-related PR loss.

## Materials and methods

### Animal subjects

All experimental procedures were approved by the Institutional Animal Care and Use Committee (IACUC) of Massachusetts Eye and Ear, and adhered to the Association for Research in Vision and Ophthalmology (ARVO) guidelines for the use of animals in ophthalmic and vision research. Mice were housed under a 12-h light/dark cycle and provided with a 4% fat rodent diet and water *ad libitum*.

To evaluate the effect of *Sting* knockout on *Nmnat1*-V9M mutation, *Nmnat1*^V9M/+^:*Sting*^+/−^ mice were bred to obtain litters containing both *Nmnat1*^V9M/V9M^:*Sting*^+/+^ and *Nmnat1*^V9M/V9M^:*Sting*^−/−^ siblings. Experiments were conducted using mice of both sexes without preference. Animals were assigned to experimental groups based on genotype and treatment. Where applicable, littermates were distributed across treatment groups to minimize potential confounding effects. No additional randomization procedures were applied.

### Genotyping

Genomic DNA was extracted from toe biopsies using the DirectPCR lysis reagent (Viagen Biotech, Los Angeles, CA, USA). The *Nmnat1* c.25G>A (p.V9M) mutation was identified via PCR amplification with primers (forward: 5′-CATGGCTGTGCTGAGGTG-3′; reverse: 5′-AACAGCCTGAGGTGCATGTT-3′) followed by Sanger sequencing (primer: 5′-ACGTATTTGCCCACCTGTCT-3′). PCR reactions were performed using Q5® High-Fidelity 2X Master Mix (New England Biolabs, Ipswich, MA, USA) under standard thermocycling conditions (98 °C for 30 s; 30 cycles of 98 °C/10 s, 68 °C/20 s, 72 °C/20 s; final extension at 72 °C/2 min) [8].

The *Sting* knockout (*Sting1*^tm1.2Camb^) allele was identified by PCR amplification using a common forward (5’-CTCCAGGAACACCGGTCTAG-3’) with either a wildtype reverse primer (5’-TGATTTGGTGGATCCTTTGC-3’) or a mutant reverse primer (5’-CGGCCGAAGTTCCTATTCTC-3’). PCR reactions were performed with BioMix™ Red 2X Master Mix (Meridian Bioscience, Cincinnati, OH, USA) under the following conditions: 95 °C for 3 min; 35 cycles of 95 °C/30 s, 58 °C/30 s, 72 °C/20 s; final extension at 72 °C/5 min.

### Drug administration

Starting at PW2, *Nmnat1*^V9M/V9M^ mice received daily intraperitoneal injections of NAC (Sigma-Aldrich, St. Louis, MO, USA) dissolved in PBS (12.5 mg/mL) at a dose of 250 mg/kg body weight for 4 weeks. Control littermates received equivalent volumes of PBS (20 mL/kg). Both NAC and PBS solutions were passed through a 0.2 µm polyethersulfone membrane filter (Thermo Fisher Scientific, Waltham, MA, USA) to remove potential contaminants before injection. The NAC dosage was selected based on previous studies demonstrating ocular protection without toxicity [34, 77, 78].

To achieve retinal microglial depletion, mice were given PLX5622-formulated (Chemgood, Henrico, VA, USA) AIN-76A chow (1200 ppm, Research Diets, New Brunswick, NJ, USA) ad libitum starting at PW3 [48–50]. Control mice were fed standard AIN-76A chow (Research Diets, New Brunswick, NJ, USA). No noticeable behavioral abnormalities or health issues were observed in mice receiving the PLX5622-supplemented diet.

### Ocular coherence tomography (OCT)

Retinal structure was assessed in NAC- and PBS-treated *Nmnat1*^V9M/V9M^ mice, as well as untreated *Nmnat1*^+/+^ controls at PW4, 5, and 6 using a Bioptigen Envisu R2200 spectral-domain OCT system (Leica Microsystems, Wetzlar, Germany). Anesthesia was induced with 4% isoflurane and maintained at 1.5–3% in 100% oxygen (0.4 L/min). Mydriasis was achieved using topical 2.5% phenylephrine (Lifestar Pharma, Mahwah, NJ, USA) and 1% tropicamide (Bausch + Lomb, Vaughan, Canada). Corneal hydration and image quality were maintained with periodic application of sterile saline (Sigma-Aldrich, St. Louis, MO, USA).

OCT imaging was performed using a linear B-scan centered on the optic nerve head with a 1.4 mm scan length, 1 000 A-scans per B-scan, and 5 B-scan averaged over 20 frames. Outer retinal thickness was measured from the inner edge of outer plexiform layer (OPL) to the outer edge of retinal pigment epithelium (RPE). Inner retinal thickness was measured from the inner edge of nerve fiber layer (NFL) to the outer edge of inner nuclear layer (INL). Retinal thickness was quantified in a masked fashion using ImageJ, with values taken from positions spanning the central to peripheral retina (–600 µm to +600 µm relative to the optic nerve head center).

### Electroretinogram (ERG)

Retinal function in the same cohort of OCT-imaged animals was assessed using the Celeris ERG system (Diagnosys LLC, Lowell, MA, USA), following previously described protocols [79]. Prior to the procedure, all mice were dark-adapted overnight (<24 h), and all manipulations were conducted under dim red illumination to preserve scotopic sensitivity. Mice were anesthetized, and mydriasis was induced as described above. Corneal hydration was maintained throughout the experiment by periodically applying lubricating eye drops containing 0.3% sterile hypromellose (Alcon Laboratories, Fort Worth, TX, USA).

For scotopic ERG, brief single white flash stimuli were delivered over a five-log unit range (−4 to 1 log cd·s/m²) to assess rod and mixed rod-cone responses. Stimuli from −4 to −1 log cd·s/m² were averaged over 20 trials, while higher-intensity stimuli (0 and 1 log cd·s/m²) were averaged over five trials. Following scotopic recordings, mice were light-adapted for 10 min under a 25 cd/m² white background to saturate rod activity. Photopic ERG responses were then recorded using single 1 Hz flash stimuli at three intensities (0, 0.48, and 1 log cd·s/m²; 4 ms duration), with 20 responses averaged per stimulus level. Additionally, cone flicker ERGs were obtained at 10 Hz under light-adapted conditions, with 30 responses averaged per frequency. For visualization, raw ERG traces were replotted in Microsoft Excel. A-wave amplitudes, reflecting PR responses, and b-wave amplitudes, reflecting bipolar cell response but dependent on the initial PR response, were manually identified using Espion E3 software (Diagnosys LLC, Lowell, MA, USA) and the resulting amplitude values were exported into GraphPad Prism 10 (GraphPad Software, Boston, MA, USA) for statistical analysis.

### Tissue processing and immunofluorescence

Tissue processing and immunofluorescence were conducted following established protocols [80]. Mice were euthanized via CO₂ asphyxiation, and eyes were enucleated and rinsed in PBS. The cornea and lens were dissected, and the posterior eyecup was fixed in 4% paraformaldehyde (PFA, v/v, Electron Microscopy Sciences, Hatfield, MA, USA) overnight at 4 °C. After PBS washes, tissues were cryoprotected in 30% sucrose (w/v) overnight at 4 °C, embedded in optimal cutting temperature compound (Sakura Finetek, Torrance, CA, USA), and sectioned at a thickness of 12 µm. For immunostaining, sections were rehydrated in PBS for 10 min, permeabilized with 0.4% Triton X-100 (Sigma-Aldrich, St. Louis, MO, USA) for 10 min, and blocked with 1% bovine serum albumin (Sigma-Aldrich, St. Louis, MO, USA) and 10% normal goat serum (Invitrogen, Waltham, MA, USA) in PBS for 45 min at room temperature. Primary antibodies were incubated overnight at 4 °C, and secondary antibodies were applied at room temperature for 2 h. Information of primary and secondary antibodies is provided in Supplementary Table 1. Retinal immune cells were labeled with anti-Iba1 antibody (rabbit host) to examine immune response dynamics. Since Iba1 and CD11b both mark microglia/macrophages in the retina, CD11b (rat host) was used instead for co-staining with STING (rabbit host).

For 8-oxo-dG detection, after permeabilization, RNA was enzymatically digested using RNase A (200 µg/mL, Thermo Fisher Scientific, Waltham, MA, USA) and RNase T1 (50 U/mL, Thermo Fisher Scientific, Waltham, MA, USA) in 140 mM NaCl (Sigma-Aldrich, St. Louis, MO, USA) for 1 h at 37 °C, followed by a 4 °C NaCl (140 mM) rinse for 5 min. DNA denaturation was performed with 70 mM NaOH for 4 min, and sections were treated with Proteinase K (20 µg/mL, Qiagen, Hilden, Germany) for 10 min at 37 °C. Residual enzymatic activity was quenched with 0.2% glycine (Sigma-Aldrich, St. Louis, MO, USA) in PBS for 10 min prior to blocking.

For mitochondrial labeling, freshly isolated eyecups were incubated with 500 nM MitoTracker® Red CMXRos diluted in phenol red-free Neurobasal A medium supplemented with B27 (2%) and N2 (1%) (all from Thermo Fisher Scientific, Waltham, MA, USA) for 30 min at room temperature. Eyecups were then fixed in 4% PFA, cryoprotected in 30% sucrose, and cryosectioned. Subsequent AIF immunostaining and Hoechst nuclear staining were performed following the standard immunostaining protocol described above.

Retinal images were captured using Nikon Eclipse Ti fluorescence microscope (Nikon, Melville, NY, USA) equipped with an Andor CCD camera (Andor Technology, Belfast, Northern Ireland) or Leica TCS SP8 confocal microscope (Leica, Wetzlar, Germany) with standardized sampling areas equidistant from the optic nerve. Positive cells were manually counted within a square-shaped area encompassing all retinal layers and normalized to 0.01 mm² according to each sample’s scale.

### TUNEL assay

TUNEL (TdT-mediated dUTP nick end labeling) assay (Thermo Fisher Scientific, Waltham, MA, USA) was performed according to the manufacturer’s protocol. Briefly, cryosections were treated with Proteinase K for 15 min at room temperature, followed by PBS washes. Sections were then incubated with the TUNEL reaction mix for 1 h at 37 °C and washed in PBS. For co-staining with 8-oxo-dG, TUNEL-labeled sections subsequently underwent immunostaining as described above. Nuclei were counterstained with Hoechst 33342 (Thermo Fisher Scientific, Waltham, MA, USA).

### Western blot

Mouse retinas were homogenized and lysed in RIPA buffer (Thermo Fisher Scientific, Waltham, MA, USA) supplemented with a protease inhibitor cocktail (Sigma-Aldrich, St. Louis, MO, USA). Protein concentrations were determined using a BCA assay (Thermo Fisher Scientific, Waltham, MA, USA). Equal amounts of protein (30 µg per sample) were separated on 4–20% precast gradient gels (Bio-Rad, Hercules, CA, USA) and transferred to PVDF membranes for 7 min using the iBlot 2 dry transfer system (Thermo Fisher Scientific, Waltham, MA, USA) with program P0. Membranes were blocked with LI-COR blocking buffer (LI-COR, Lincoln, NE, USA) for 2 h at room temperature, then incubated with primary and secondary antibodies. Protein signals were detected using the Odyssey imaging system (LI-COR, Lincoln, NE, USA). Details of antibodies used are provided in the Supplementary Table 1.

### Malondialdehyde (MDA) assay

MDA levels in mouse retinas were measured using a thiobarbituric acid-based method following the MDA Colorimetric Assay Kit protocol (Thermo Fisher Scientific, Waltham, MA, USA). Briefly, freshly collected retina tissue (two retinas from one mouse pooled as a single sample) was homogenized in 100 µL of PBS. After centrifugation, 40 µL of the supernatant was used for sample duplicates (20 µL each), while the remaining supernatant was used to determine protein concentration using the BCA assay (Thermo Fisher Scientific, Waltham, MA, USA). Following the reaction with thiobarbituric acid, the optical density of each sample and standard was measured at 532 nm using a SpectraMax plate reader and SoftMax Pro software (Molecular Devices, San Jose, CA, USA). MDA concentrations were calculated using the excel template provided on the manufacturer’s website.

### NAD^+^ assay

NAD⁺ levels in mouse retinas were measured using an enzyme cycling-based colorimetric method (NAD/NADH Assay Kit, Abcam, Waltham, MA, USA) following the manufacturer’s protocol. Briefly, mouse retinas were snap-frozen and stored in liquid nitrogen for less than one week until all samples were ready for analysis. Two retinas from a single mouse were pooled as one sample. Reactions were performed according to the manufacturer’s instructions. Samples were loaded in duplicate with a 1:5 dilution. After 2 h of incubation, the optical density of each sample and standard was measured at 450 nm using a SpectraMax plate reader and SoftMax Pro software (Molecular Devices, San Jose, CA, USA). NAD⁺ concentrations were then calculated in Excel using the equation provided in the kit protocol.

### Mouse blood collection and comprehensive metabolic panel test

To assess liver and kidney function, terminal blood collection was performed in mice at 6 weeks of age. Immediately after euthanasia, the thoracic cavity was opened to expose the heart, and blood was collected via cardiac puncture using a 25-gauge needle inserted at the xiphoid process at a shallow angle. Approximately 400–600 µL of whole blood was collected per mouse. Blood samples were transferred to Microtainer blood collection tubes (Becton Dickinson, Franklin Lakes, NJ, USA) and allowed to clot at room temperature for 30 min. Samples were then transported to the Massachusetts General Hospital Center for Comparative Medicine for serum isolation and Comprehensive Metabolic Panel analysis. All analyses were conducted according to standard clinical chemistry protocols at the core facility.

### Statistics

Data were analyzed using Student’s *t* tests or ANOVA with a significance threshold of α = 0.05. Results are presented as mean ± standard deviation. Detailed statistical parameters, including sample sizes and replicates, are provided in the figure legends. All analyses were performed using GraphPad Prism 10 (GraphPad Software, Boston, MA, USA). No animals were excluded from the analysis. Sample sizes were based on previous published studies, and no formal a priori power calculation was performed.

## Supplementary information

Supplementary Material

Original uncropped Western Blot image

## Data Availability

All data relevant to the study are included in the article or uploaded as supplementary information. Source data are available upon reasonable request.
