# Supplementary material for: Oxidative DNA damage drives apoptotic photoreceptor loss in NMNAT1-associated inherited retinal degeneration: a therapeutic opportunity
Source: Cell Death Dis. 2026 Apr 2;17(1):442. doi: 10.1038/s41419-026-08680-7 (PMC13168578; doi:10.1038/s41419-026-08680-7)
Supplement: Supplementary file 1 — Supplementary Material [file 41419_2026_8680_MOESM1_ESM.docx]

**Supplementary Material**

**Supplemental Table 1.** List of primary and secondary antibodies used in experiments.

1. **Primary Antibody**

| **Antigen** | **Host Species** | **Catalog #** | **Dilution** | **Source** |
| --- | --- | --- | --- | --- |
| 8-oxo-dG | Mouse | 4354MC050 | 1:200 | R&D Systems |
| γH2A.X | Rabbit | 4418-APC-100 | 1:200 | Bio-Techne |
| GFAP | Rabbit | AB68428 | 1:200 | Abcam |
| Iba1 | Rabbit | 019-19741 | 1:150 | Fujifilm |
| CD11b | Rat | 50-124-12 | 1:100 | Thermo Fisher Scientific |
| Caspase-9 | Rabbit | AB52298 | 1:200 | Abcam |
| AIF | Rabbit | AB1998 | 1:100 | Abcam |
| p-MLKL | Rabbit | AB196436 | 1:200 | Abcam |
| STING | Rabbit | 50494 | 1:100 for immunofluorescence; 1:1 000 for western blot | Cell Signaling |
| γ-tubulin | Mouse | T6557 | 1:1 000 | Sigma-Aldrich |
| 4-HNE | Rabbit | HNE11-S | 1:100 | Alpha Diagnostic International |
| MitoTracker | N/A | M7512 | 5 µM | Thermo Fisher Scientific |

1. **Secondary Antibody**

| **Antigen** | **Catalog #** | **Dilution** | **Source** |
| --- | --- | --- | --- |
| Goat-anti-mouse Alexa Fluor 488 | A28175 | 1:1 000 | Thermo Fisher Scientific |
| Goat-anti-rabbit Alexa Fluor 555 | A21428 | 1:1 000 | Thermo Fisher Scientific |
| Goat-anti-rabbit Alexa Fluor 488 | A11008 | 1:1 000 | Thermo Fisher Scientific |
| Goat-anti-rat Alexa Fluor 647 | A21247 | 1:1 000 | Thermo Fisher Scientific |
| Hoechst 33342 | H3570 | 1:1 000 | Thermo Fisher Scientific |
| IRDye 800CW goat anti-rabbit IgG | 926-32211 | 1:20 000 | LI-COR |
| IRDye 680RD goat anti-mouse IgG | 926-68070 | 1:20 000 | LI-COR |

**
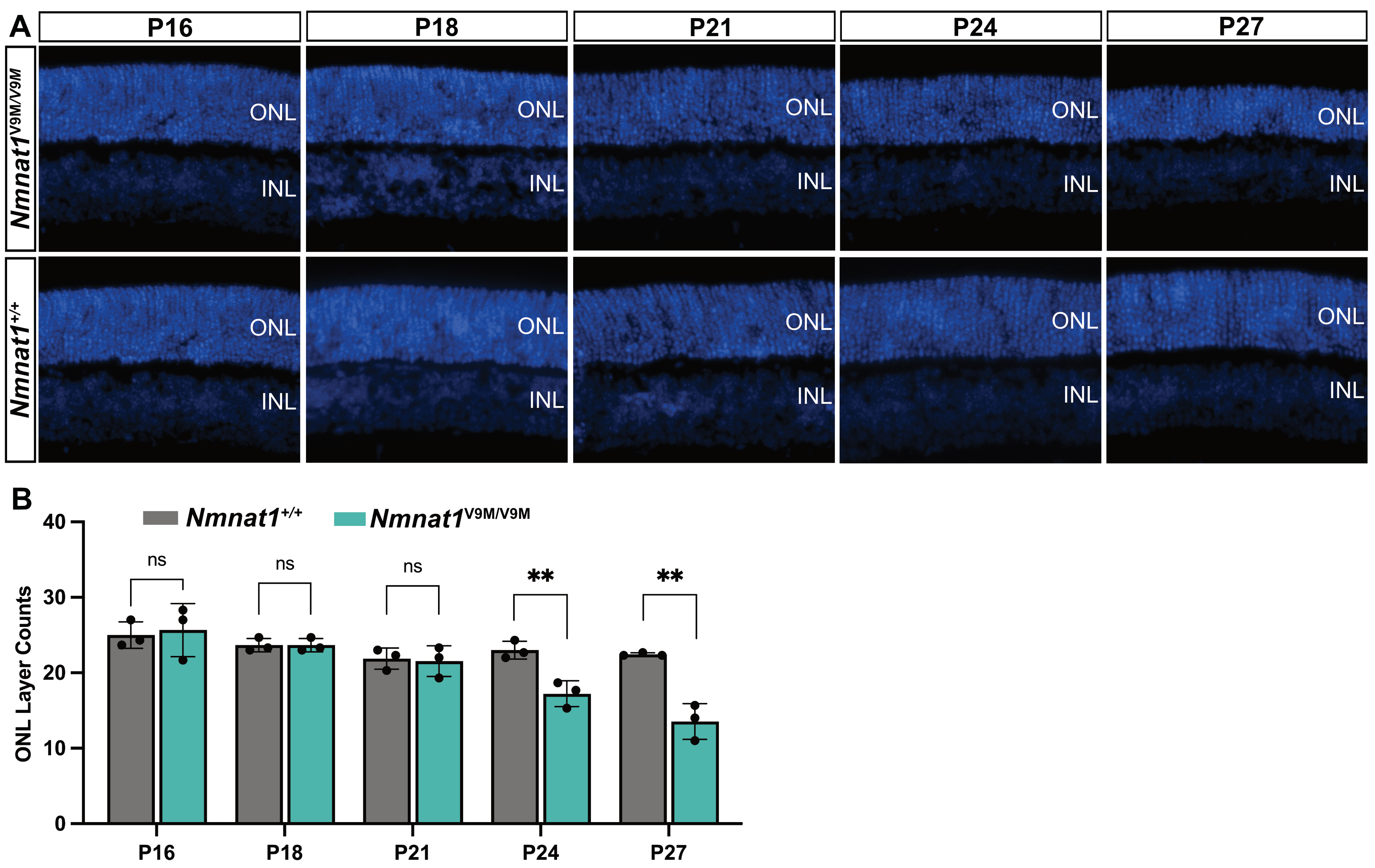
**

**Supplemental Figure 1. PR loss in *Nmnat1*^V9M/V9M^ mouse retinas from P18-P27.**

(A) Representative images of Hoechst-stained retinas from *Nmnat1*^V9M/V9M^ and *Nmnat1*^+/+^ mice at P18-P27. (B) Quantification of Hoechst-positive nuclear layers in the ONL across P18-P27. Three measurements per retina were averaged for each replicate, and comparisons between *Nmnat1*^V9M/V9M^ and *Nmnat1*^+/+^ groups were performed at each time point using unpaired *t*-tests. Measurements were performed using Fiji with the MTB Cell Counter. Data are presented as mean ± standard deviation*. n* = 3 biological replicates per group. ****p* < 0.001, ***p* < 0.01, **p* < 0.05; ns, non-significant.

**
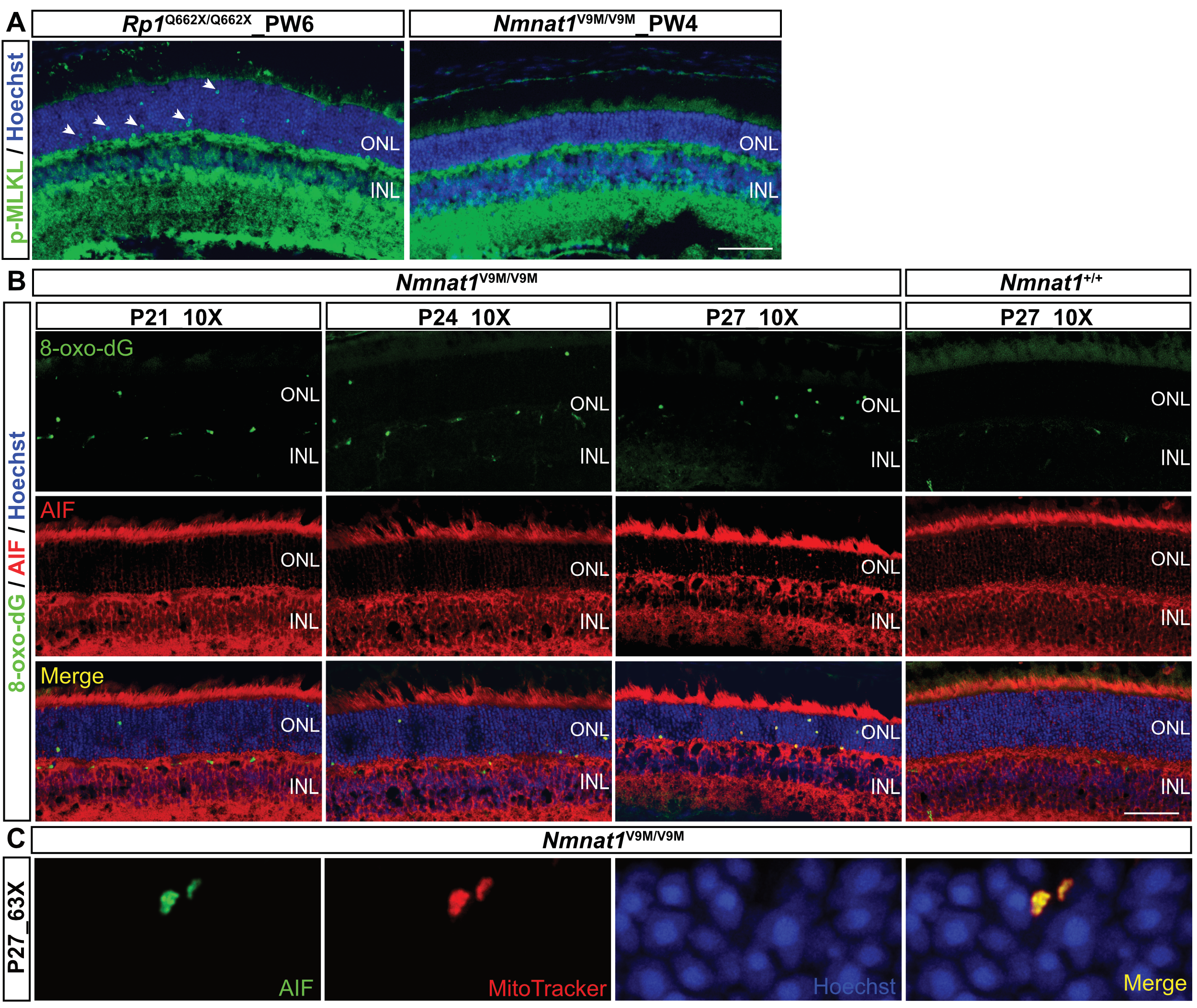
**

**Supplemental Figure 2. Necroptosis and parthanatos contribute minimally to *Nmnat1*-associated retinal degeneration.** (A) Immunofluorescence analysis of the necroptosis marker phospho-MLKL (green) in the *Nmnat1*^V9M/V9M^ retina at PW4 shows no detectable signal, indicating the absence of necroptosis during PR degeneration. *Rp1*^Q662X/Q662X^ mice at PW6, which undergo PR degeneration, were used as a positive control to confirm the reactivity of the phospho-MLKL antibody. Nuclei are counterstained with Hoechst (blue). (B) Immunofluorescence of AIF (red), a marker of parthanatos, reveals its presence at later time points (P24 and P27) in the *Nmnat1*^V9M/V9M^ mouse retina. Scale bar = 100 µm. (C) High-magnification (63X, zoom factor = 10) images show that AIF (green) colocalizes with MitoTracker (red) but does not translocate into Hoechst-labeled nuclei (blue).


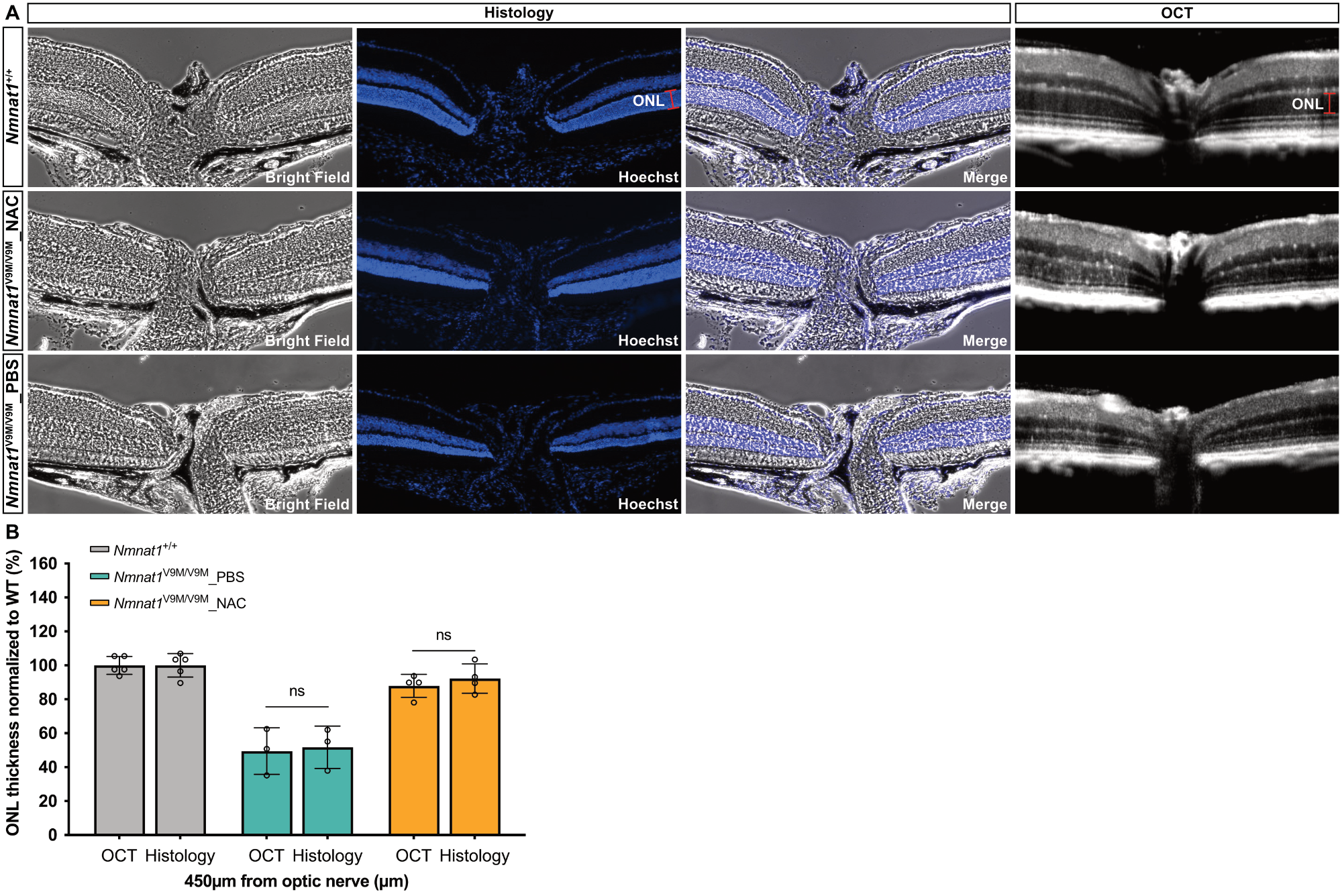


**Supplemental Figure 3. Histological verification of OCT measurements.**

(A) Representative histology and OCT images of *Nmnat1*^+/+^ and NAC- or PBS-treated *Nmnat1*^V9M/V9M^ mouse retinas at PW6. (B) Quantification of ONL thickness at 450 µm from the center of the optic nerve head in PBS-treated (green) and NAC-treated (orange) *Nmnat1*^V9M/V9M^ retinas, normalized to *Nmnat1*^+/+^ control (100%, gray) in both OCT and histology images to minimize potential artifacts introduced during histological processing. Normalized ONL thickness values obtained from OCT and histology were highly consistent, with no significant differences between the two methods (unpaired *t*-tests), confirming the reliability of OCT measurements in assessing PR preservation/loss. *n* = 3-5 mice per group. Data are presented as mean ± standard deviation.

**
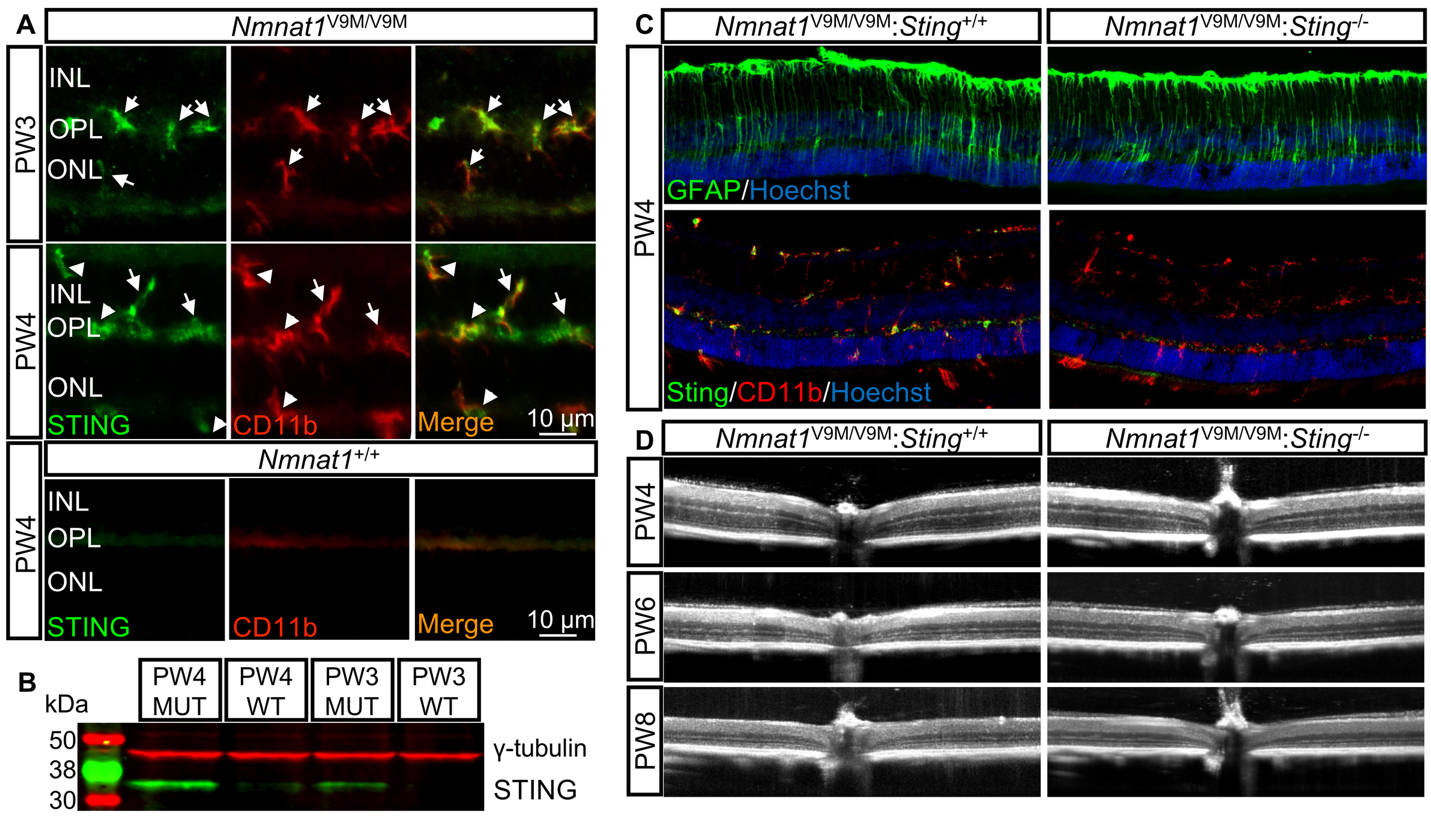
Supplemental Figure 4. Activation of the cGAS-STING pathway and its limited contribution to retinal immune response in *Nmnat1*^V9M/V9M^ mouse retina.**(A) Immunofluorescence images showing STING (green), CD11b (red), and Hoechst (blue) staining in *Nmnat1*^V9M/V9M^ mouse retina at PW3 and PW4. (B) Western blot analysis of STING (35kDa) and the housekeeping protein γ-tubulin (48kDa) in retinal protein extracts from *Nmnat1*^+/+^ (WT) and *Nmnat1*^V9M/V9M^ (MUT) mice. (C) Immunofluorescence images of GFAP (green, upper panel), and STING (green, bottom panel) and CD11b (red, bottom panel) in *Nmnat1*^V9M/V9M^:*Sting*^+/+^ and *Nmnat1*^V9M/V9M^: *Sting*^-/-^ mouse retinas at PW4. (D) OCT imaging of *Nmnat1*^V9M/V9M^:*Sting*^+/+^ and *Nmnat1*^V9M/V9M^:*Sting*^-/-^ mouse retinas from PW4 to PW8.


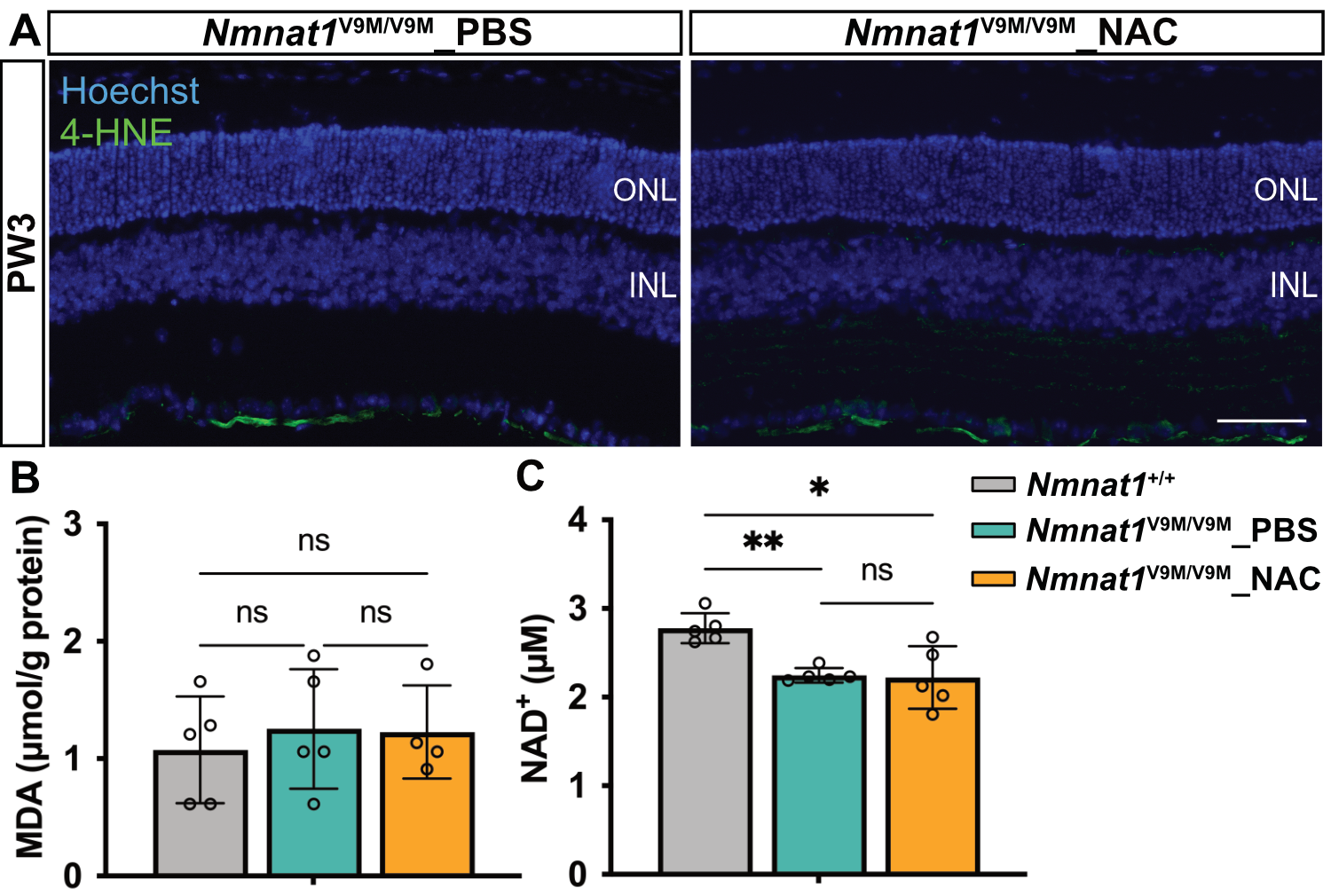


**Supplemental Figure 5. Effects of NAC on lipid peroxidation and NAD⁺ levels.**
(A) The lipid peroxidation marker 4-hydroxynonenal (4-HNE) was not detected in retinas from PBS- or NAC-treated *Nmnat1*^V9M/V9M^ mice. (B) No significant differences were observed in levels of another lipid peroxidation marker, malondialdehyde (MDA), among *Nmnat1*^+/+^ mice and PBS- or NAC-treated *Nmnat1*^V9M/V9M^ mice. (C) *Nmnat1* mutation causes a significant reduction in retinal NAD⁺ levels compared with *Nmnat1*^+/+^ controls, and NAC treatment does not restore NAD⁺ levels in *Nmnat1*^V9M/V9M^ mouse retinas. Statistical comparisons between groups were performed using one-way ANOVA followed by Tukey’s post hoc multiple comparisons test. Data are presented as mean ± standard deviation*. n* = 5 mice for each group. ***p* < 0.01, **p* < 0.05; ns, non-significant. Scale bar = 100 µm.


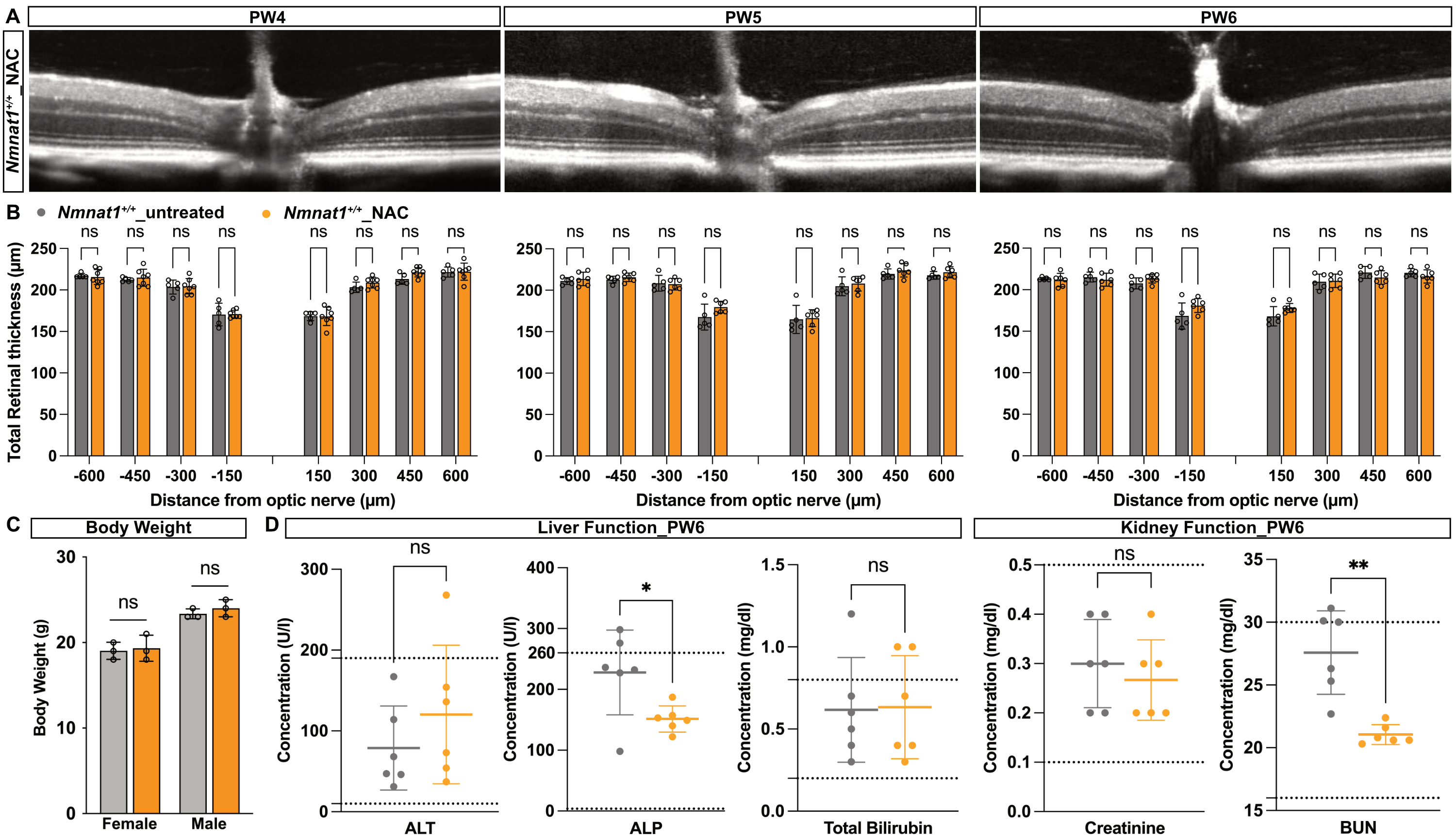


**Supplemental Figure 6. Safety profile of NAC treatment in *Nmnat1*^+/+^** **mice.**

(A) Representative OCT images of retinas from NAC-treated *Nmnat1*^+/+^ mice from PW4 to PW6. No structural abnormalities were observed. (B) Quantification of total retinal thickness in untreated *Nmnat1*^+/+^ (gray) and NAC-treated *Nmnat1*^+/+^ mice (orange). No significant differences were detected at any time point across positions from −600 µm to +600 µm relative to the center of the optic nerve head. (C) Body weight measurements of female (gray) and male (orange) *Nmnat1*^+/+^ mice after 4 weeks of NAC treatment (PW6, orange) show no significant changes compared with untreated controls (gray). (D) Serum parameters reflecting liver and kidney function show no significant differences between NAC-treated (orange) and untreated (gray) *Nmnat1*^+/+^ mice. Dashed lines indicate the normal reference ranges. *ALT*, alanine aminotransferase; *ALP*, alkaline phosphatase; *BUN*, blood urea nitrogen. Statistical comparisons between groups were performed using *t*-tests. Data are presented as mean ± standard deviation. For OCT measurements*, n* = 5 for untreated *Nmnat1*^+/+^ mice group and n = 6 for NAC-treated *Nmnat1*^+/+^ mice group. For body weight and blood tests, *n* = 6 per group. ***p* < 0.01, **p* < 0.05; ns, non-significant.
