# Supplementary material for: Oxidative DNA damage drives apoptotic photoreceptor loss in NMNAT1-associated inherited retinal degeneration: a therapeutic opportunity
Source: Cell Death Dis. 2026 Apr 2;17(1):442. doi: 10.1038/s41419-026-08680-7 (PMC13168578; doi:10.1038/s41419-026-08680-7)
Supplement: Supplementary file 2 — Original uncropped Western Blot image [file 41419_2026_8680_MOESM2_ESM.docx]

**
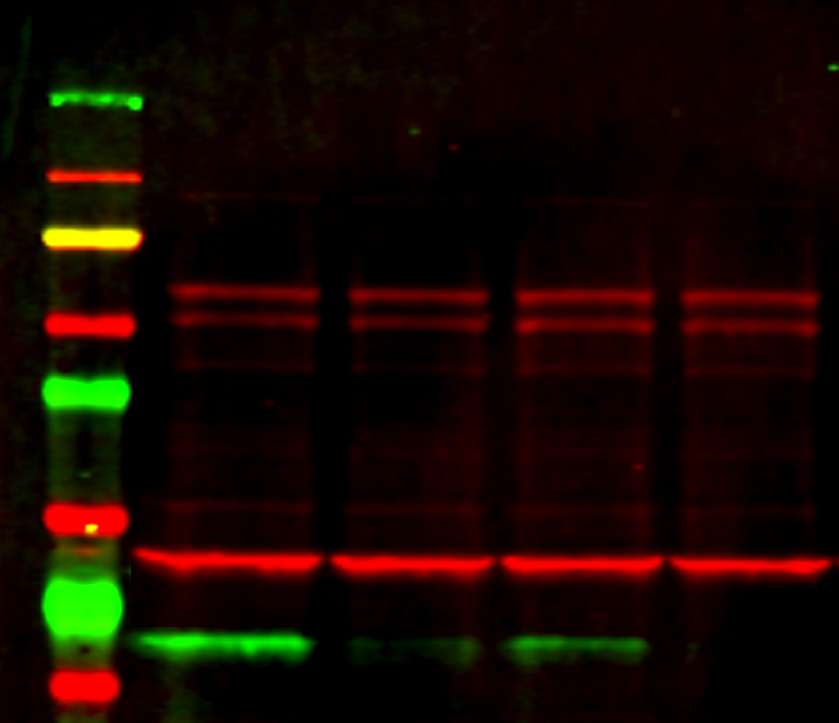
**

**Original uncropped Western Blot image.**

Uncropped Western Blot image showing protein expression detected with antibodies against γ-tubulin, STING, and Phospho-Stat3 (not shown in the main figures of this study).
